# Pre-clinical Studies Identifying Molecular Pathways of Neuroinflammation in Parkinson's Disease: A Systematic Review

**DOI:** 10.3389/fnagi.2022.855776

**Published:** 2022-07-04

**Authors:** Mobina Fathi, Kimia Vakili, Shirin Yaghoobpoor, Mohammad Sadegh Qadirifard, Mohammadreza Kosari, Navid Naghsh, Afsaneh Asgari taei, Andis Klegeris, Mina Dehghani, Ashkan Bahrami, Hamed Taheri, Ashraf Mohamadkhani, Ramtin Hajibeygi, Mostafa Rezaei Tavirani, Fatemeh Sayehmiri

**Affiliations:** ^1^Student Research Committee, Faculty of Medicine, Shahid Beheshti University of Medical Sciences, Tehran, Iran; ^2^Department of Nursing and Midwifery, Islamic Azad University, Tehran, Iran; ^3^Department of Nursing, Garmsar Branch, Islamic Azad University, Garmsar, Iran; ^4^The First Clinical College, Wuhan Union Hospital, Tongji Medical College, Huazhong University of Science and Technology, Wuhan, China; ^5^Department of Pharmacy, Shahid Sadoughi University of Medical Sciences, Yazd, Iran; ^6^Neuroscience Research Center, Shahid Beheshti University of Medical Sciences, Tehran, Iran; ^7^Department of Biology, Faculty of Science, University of British Columbia Okanagan Campus, Kelowna, BC, Canada; ^8^School of Medicine, Isfahan University of Medical Sciences, Isfahan, Iran; ^9^Faculty of Medicine, Kashan University of Medical Science, Kashan, Iran; ^10^Dental School, Kazan Federal University, Kazan, Russia; ^11^Digestive Disease Research Center, Tehran University of Medical Sciences, Tehran, Iran; ^12^Department of Cardiology, Faculty of Medicine, Tehran Medical Sciences, Islamic Azad University, Tehran, Iran; ^13^Proteomics Research Center, Faculty of Paramedical Sciences, Shahid Beheshti University of Medical Sciences, Tehran, Iran

**Keywords:** Parkinson's disease, nuclear factor *kappa* B (NF-κB), NLRP3 inflammasome, microglia, mast cells, neuroinflammation

## Abstract

Parkinson's disease (PD), the second most common neurodegenerative disorder, is characterized by neuroinflammation, formation of Lewy bodies, and progressive loss of dopaminergic neurons in the substantia nigra of the brain. In this review, we summarize evidence obtained by animal studies demonstrating neuroinflammation as one of the central pathogenetic mechanisms of PD. We also focus on the protein factors that initiate the development of PD and other neurodegenerative diseases. Our targeted literature search identified 40 pre-clinical *in vivo* and *in vitro* studies written in English. Nuclear factor *kappa* B (NF-kB) pathway is demonstrated as a common mechanism engaged by neurotoxins such as 1-methyl-4-phenyl-1,2,3,6-tetrahydropyridine (MPTP) and 6*-*hydroxydopamine (6-OHDA), as well as the bacterial lipopolysaccharide (LPS). The α-synuclein protein, which plays a prominent role in PD neuropathology, may also contribute to neuroinflammation by activating mast cells. Meanwhile, 6-OHDA models of PD identify microsomal prostaglandin E synthase-1 (mPGES-1) as one of the contributors to neuroinflammatory processes in this model. Immune responses are used by the central nervous system to fight and remove pathogens; however, hyperactivated and prolonged immune responses can lead to a harmful neuroinflammatory state, which is one of the key mechanisms in the pathogenesis of PD.

## Graphical Abstract

Servier (2022). 
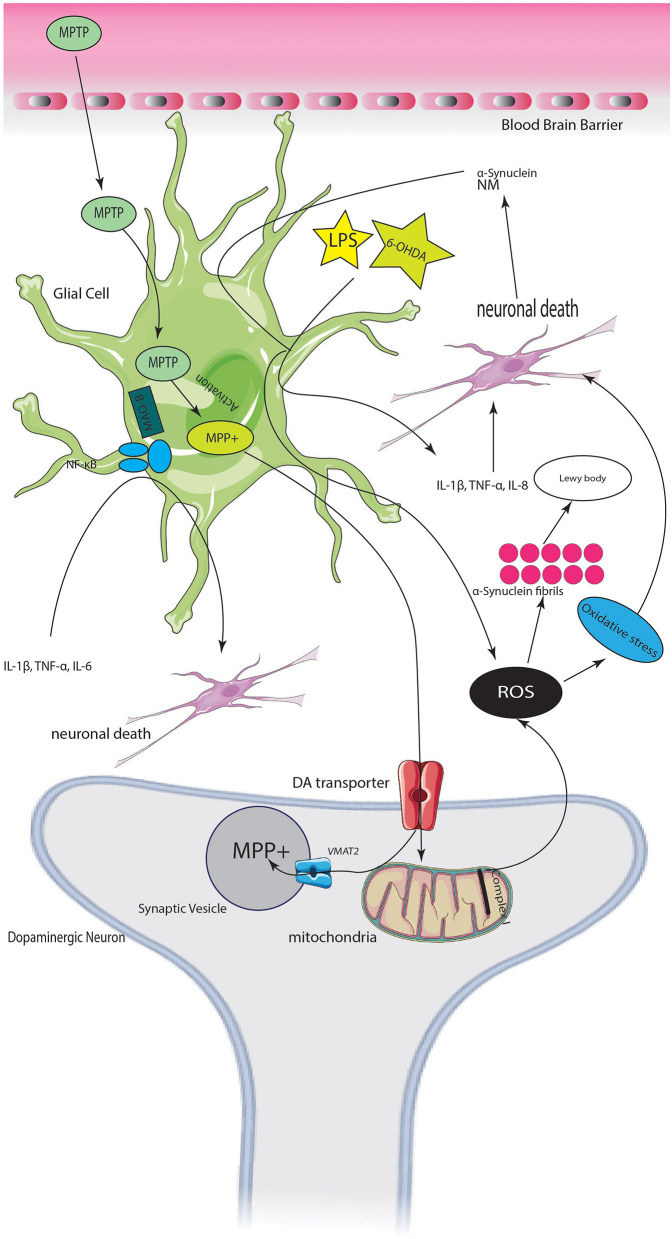

## Introduction

Parkinson's disease (PD) is a neurodegenerative disorder defined by dopaminergic neuronal loss in the substantia nigra (SN) pars compacta, aggregation of misfolded α-synuclein (α-syn) as Lewy bodies, and motor dysfunction (Trudler et al., 2015). Degeneration of the nigrostriatal pathway leads to hallmark motor symptoms of this disease, including bradykinesia, rigidity, tremor at rest, and postural instability. Furthermore, most patients exhibit non-motor symptoms, such as sleep disorders, autonomic nervous system dysfunction, and cognitive impairment (Trudler et al., 2015; Kempuraj et al., 2016). Currently, pharmacological and surgical interventions are the main treatment options in PD which provide only symptomatic relief for patients. Among various disease-specific pathogenetic mechanisms, neuroinflammation plays a prominent role in the onset and progression of a broad range of neurodegenerative disorders, including PD, Alzheimer's disease (AD), and multiple sclerosis (MS). It has been demonstrated that the sterile neuroinflammation in neurodegenerative diseases embodies a cascade of events involving abnormal protein aggregates, upregulated inflammatory mediators, and activation of non-neuronal glial cells, which leads to neuronal damage. Neurodegeneration, in turn, induces further glial activation and neuroinflammation in the central nervous system (CNS). Out of the several different non-neuronal cell types of the CNS, microglia are the most prominent contributors to neuroimmune reactions. They express several different pattern recognition receptors (PRRs), such as toll-like receptor (TLR)2, TLR4, TLR9, and receptor for advanced glycation end products (RAGE), that recognize various pathogens and abnormal proteins. Microglial activation leads to the secretion of various pro-inflammatory mediators, including interleukin (IL) 1β, IL-6, and tumor necrosis factor α (TNF-α), to restore tissue hemostasis and also facilitate tissue repair (Ransohoff and Brown, 2012; Ransohoff, 2016; Molteni and Rossetti, 2017). Astrocytes represent another type of glial cells, which are critical for neuronal networks and maintenance of brain tissue homeostasis. Similar to microglia, astrocytes express PRRs and can contribute to neuroimmune responses by releasing a broad range of inflammatory mediators (Cunningham et al., 2019). Although acute inflammatory response can clear abnormal proteins, eliminate cell debris, and promote tissue repair, persistent inflammation is detrimental since it produces harmful inflammatory mediators and cytotoxins, as well as inhibits neural regeneration.

Neuroinflammation contributes to PD pathogenesis throughout the progression of this disease from early α-syn aggregation to causing dopaminergic cell loss and ultimately PD symptoms. For example, the accumulation of misfolded α-syn has been suggested to cause dysregulation of both innate and adaptive immune responses (Harms et al., 2021). Strong support to the neuroinflammatory hypothesis of PD is provided by genome-wide association studies linking sporadic PD to polymorphism in the human leukocyte antigen (HLA) region containing HLA-DR gene (Mohamadkhani et al., 2009; Simón-Sánchez et al., 2009; Hamza et al., 2010; Ahmed et al., 2012). The sustained inflammatory responses have been described in both PD patients and animal models of this disease, which through various mechanisms can cause neuronal dysfunction. In addition to reactive microglia and astrocytes releasing neurotoxic molecules, other mechanisms contributing to neuronal death and neurodegeneration in PD have been discovered; they include brain infiltration and activation of inflammatory mast cells and T lymphocytes, increased oxidative stress, and upregulation of inflammatory signaling molecules (Lyman et al., 2014; Jarrott and Williams, 2016; Kempuraj et al., 2016).

Activated glial cells and infiltrating peripheral immune cells are the main sources of the various pro-inflammatory mediators contributing to the onset and progression of PD. Activated glial cells release a broad range of both pro- and anti-inflammatory cytokines, such as TNF-α, IL-1β/IL-1α, IL-6, IL-8, and IL-10, as well as the brain inflammatory protein called glia maturation factor (GMF), which regulates functions of glial cells and can also induce neurodegeneration in the brain (Lim et al., 2004; Zaheer et al., 2008a,b; Tore and Tuncel, 2009; Kempuraj et al., 2013; Molteni and Rossetti, 2017; Mukai et al., 2018). Activated mast cells release potentially harmful mediators, such as proteases, utilizing the degranulation process (Tore and Tuncel, 2009; Taracanova et al., 2017; Mukai et al., 2018).

Dopaminergic neurotoxins such as 6-OHDA, 1-methyl-4-phenyl-1,2,3,6-tetrahydropyridine (MPTP), and its metabolite 1-methyl-4-phenylpyridinium (MPP^+^) have been shown to adversely alter neuronal functions in both mature and developing nervous tissue. They have been used to induce PD in animal models where they, in addition to dopaminergic neuronal damage, cause glial activation, oxidative stress, mitochondrial damage, and the release of inflammatory cytokines (Członkowska et al., 1996; Stojkovska et al., 2015; Trudler et al., 2015; Pourasgari and Mohamadkhani, 2020). *In vivo* and *in vitro* studies show that the mechanism of action for dopaminergic neurotoxins could involve high-mobility group box 1 (HMGB1) (Huang et al., 2017), which is a nuclear DNA binding non-histone protein that facilitates the assembly of nucleoprotein complexes, but can also be released extracellularly and act as a damage-associated molecular pattern (DAMP) triggering neuroimmune responses (Sims et al., 2010).

The present systematic review aims to identify and evaluate the underlying genes and mechanisms of neuroinflammation in PD pre-clinical studies. Following this, a gene list-independent approach to compare different disease models is used.

## Method

### Search Strategy

Electronic databases PubMed, Scopus, Google Scholar, Web of Science, and EMBASE were searched using the medical subject headings (MeSH) aimed at identifying all research articles related to the topic “The association between neuroinflammation and Parkinson's disease.” Two authors independently conducted the search using search strategies specific for each database and reviewed all relevant peer-reviewed articles published before April 2022. The following search terms were used “[(neuro-inflammation) AND Parkinson's disease],” “[(neuro-inflammation) AND neurodegenerative disease],” “[((inflammation) AND Parkinson's disease) AND brain],” “(adaptive immunity) AND (Parkinson), (immunological biomarkers) AND (Parkinson), (hHumoral immunity) AND (Parkinson),” “[((inflammation) AND neurodegenerative disease) AND brain],” “[(inflammatory markers) AND Parkinson's disease],” and “[(inflammatory markers) AND neurodegenerative disease],” ”[((inflammation) AND Parkinson's disease) AND animal].” A total of 40 articles were included.

### Types of Studies (Selection Criteria)

This review article considered both quantitative and qualitative data on the association between neuroinflammation and PD, which were obtained by reviewing all available *in vivo* and *in vitro* pre-clinical studies relevant to this topic. Review articles and studies with human subjects were excluded. Only articles published in English were included. Duplicate data and low-quality studies, identified by the Systematic Review Center for Laboratory animal Experimentation (SYRCLES) quality assessment checklist (Hooijmans et al., 2014), were excluded. All eligible studied, both with positive and negative findings have been included in this systematic review. SYRCLES quality assessment checklist (Hooijmans et al., 2014) was used to assess selection bias, performance bias, detection bias, attrition bias, reporting bias, and other sources of bias (see Supplementary Table 1).

### Data Extraction

The relevant studies were selected after the title, abstract, and full-text screening of the articles. In addition, the reference lists of selected studies were reviewed to identify any additional articles, should they have not been identified by the search process. The following information was extracted from each of the identified studies: cell culture and animal model used, the size of the PD model and control groups, inflammatory markers studied, and the key results obtained (see Table 1). This study was approved by the Iranian National Committee for Ethics in Biomedical Sciences (Code of Ethics: IR.SBMU.RETECH.REC.1399.992).

**Table 1:** A rapid review of articles assessed.

**References**	**Type of *in vitro*/*in vivo* models**	**Age and sex of animal models**	**Experimental design**	**Method used to induce PD**	**Type of immune cell**	**Inflammatory factors**	**Disease mechanism**	**New therapeutic targets**
Zhang et al. (2022)	N9 microglial cells C57BL/6 mice	Age and sex not specified	*In vivo/In vitro*	LPS	Microglia	NLRP3, caspase-1, tumor necrosis factor-α (TNF-α), IL-1β and inducible nitric oxide synthase (iNOS)	AMS-17 inhibits NLRP3 pathways and microglial activation.	
Karikari et al. (2022)	Wildtype C57BL/6 or Recombination-activating gene 1 (RAG1)^−/−^ mice		*In vivo* stereotactically injection of Adeno-associated Vector 1/2 serotype (AAV1/2) containing A53T α-syn to the SN	AAV-A53T- α-syn	B cells		B cells do not protect neurons from neurodegeneration	
Lai et al. (2021)	Young adult wild-type mice (C57BL/6N)	2.5-month-old Male	*In vivo* The unilateral intracranial injection	PFF α-syn–injected	Microglia	α-syn	Neuroinflammation may occur before α-syn pathologic aggregation	
La Vitola et al. (2021)	C57BL/6 naive mice and A53T α-synuclein transgenic PD mice	8-week-old C57BL/6 naive mice 8-months-old A53T mice Sex not specified	*In vivo*	LPS	Microglia and astrocytes	α-syn	Peripheral neuroinflammation induces PD by α-syn accumulation and causes motor deficits in A53T mice	
Mao et al. (2021)	PC12 cells	–	*In vitro*	LPS	Microglia	Lipoic acid	Lipoic acid inhibits p53/NF–κB pathway	
Zhang et al. (2021)	*In vitro*: BV2 murine microglial cells Human embryonic kidney (HEK) 293T cells Rat primary microglia extracted from the brains of Sprague-Dawley rats *In vitvo*: Sprague-Dawley rats	0 or 1-day-old Male	*In vitro/in vivo* stereotaxic injection of LPS	LPS	Microglia	α-syn metabotropic glutamate receptor 5 (mGluR5)	α-syn causes lysosome-dependent degradation of mGluR5 to occur faster. mGluR5 regulates neuroinflammation	
Williams et al. (2021)	C57BL/6 (wild-type), *Tcrb*^−/−^, *Cd*4^−/−^, and *Cd*8^−/−^ mice	Sex and age not specified	*In vivo* stereotaxic surgery	The α-synuclein overexpression mouse model, T cell deficiency	Myeloid cells CD4 and CD8 T cells	α-syn major histocompatibility complex II (MHCII) IFNγ	α-syn causes the major histocompatibility complex II (MHCII) protein on CNS myeloid cells to be upregulated and leads to recruitment of CD4 and CD8 T cells into the CNS. These cells produce IFNγ	
Trudler et al. (2021)	Human induced pluripotent stem cell (hiPSC)-derived microglia (hiMG) hiMG with αSyn in humanized mouse brain	Sex and age not specified	*In vitro/In vivo* stereotactically injection of hiMG	A53T α-syn secreted from hiPSC-derived A9-DA neurons	Microglia	α-syn antibody Caspase 1 TLR2	In vitro, NLRP3) inflammasome is activated by α-syn through TLR2 leading to interleukin-1β secretion. αSyn–antibody complexes had a positive effect on this inflammation process. In vivo, α-syn antibody worsened caspase-1 activation and neurotoxicity	
Huang et al. (2020)	SH-SY5Y cells	–	*In vitro* plated in 10 cm dishes with various doses of PQ added	Paraquat		HMGB1	Paraquat induces an increase of HMGB1 which results in cytokine release through RAGE-P38-NF-κB signaling pathway	Knockdown of HMGB1
Sarkar et al. (2020a)	C57/BL mice Primary microglial cells from postnatal mouse pups	Sex and age not specified	*In vitro/In vivo*	αSyn_Agg_ pre-formed fibrils	Microglia	αSyn_Agg_	αSyn_Agg_ causes downregulation of progranulin (*Grn*) gene in microglia demonstrating immune roles of this gene in PD	
Subbarayan et al. (2020)	T cell deficient (athymic nude) and T cell competent (heterozygous) rats	Sex and age not specified	*In vivo* adeno-associated virus (AAV) α-syn	AAV9-α-syn	CD4+ and CD8+ T cells, micoglia		CD4+ and CD8+ T cells cause overexpression of MHCII in microglia and DA cell loss	
Iba et al. (2020)	α-syn transgenic (tg) mice (e.g., Thy1 promoter line 61)	10–11 months old Sex not specified	*In vivo*	α-syn	CD3+/CD4+ T cells		CD3+/CD4+ T cells recruited into the brain in synucleinopathies	
Morales-Garcia et al. (2020)	SH-SY5Y Cell Culture, Primary rat ventral mesencephalic neuron/glia cultures, male Wistar rats	Age not specified	*In vitro/In vivo* Injection of LPS into the right side of the SN.	6-OHDA LPS		PDE7	An elevated level of PDE7 happens due to oxidative stress caused by 6-OHDA or LPS.	
Sarkar et al. (2020b)	Primary mouse microglia	–	*In vitro*	Aggregated αSyn stimulation	Microglia	Kv1.3: a voltage-gated potassium channel	Fyn kinase mediates Kv1.3 channels. Expression of Kv1.3 channels increases	
Javed et al. (2020)	GMF knockout (GMF^−/−^) and C57BL/6 wild-type (WT) mice	Sex and age not specified	*In vivo*	MPTP: 1-methyl-4-phenyl-1,2,3,6-tetrahydropyridine		GMF IL-1β and IL-18	GMF causes NLRP3 inflammasome inhibition and decrease in the level of IL-1β and IL-18	
Li Y. et al. (2019)	A53T mice, Murine microglial cell line BV-2 and murine RAW 264.7 cell line, B Brain tissue from Neonatal and 3-month-old mice	Brain tissue from 3-months-old mice Sex not specified	*In vivo*/*in vitro* Stereotaxic surgery	α-syn	Microglia	α-syn CXCL12	α-syn induces CXCL12 upregulation and its release from microglia through TLR4/IκB-α/NF-κB pathway. Microglia migrate to the SN as a result	
Kempuraj et al. (2019)	Mouse bone marrow-derived mast cells (BMMCs), astrocytes of C57BL/6 fetal mice.	–	*In vitro*	MPTP	Astrocytes, Mast cells, Microglia	Interleukin-33 ERK1/2 MAPKs NF-κB GMF p38	Astrocytes and glia-neurons affected by mast cell proteases and mast cells affected by GMF and MPP+ are activated through ERK1/2 MAPKs and NF-κB pathways and release IL-33 MPTP-induced mast cells secrete CCL2 and express UDP4 which are both significant in the pathogenesis of PD	Abrogation of ERK1/2 MAPKs and NF-κB pathways in mast cells, glia-neurons, and astrocytes
Kempuraj et al. (2016)	Primary mouse bone marrow-derived mast cells (BMMCs), mouse primary astrocyte culture	–	*In vitro*	MPTP	Mast cells	CCL2 UCP4	MPTP-induced mast cells secrete CCL2 and express UDP4 which are both significant in the pathogenesis of PD	
Ikeda-Matsuo et al. (2019)	mPGES-1 knockout mice, Male and female wild-type mice primary mesencephalic culture generated from pregnant mPGES-1 KO and WT mice at E15	Male Age not specified Female Age not specified	*In vivo/In vitro* Administration of the neurotoxin locally and unilaterally	6-OHDA		mPGES-1 PGE2	6-OHDA induces PD in mice and causes expression of mPGES-1 which subsequently results in PGE2 production and neural cell loss in the SN	
Cao et al. (2012)	Primary microglial cultures from wild-type mice	–	*In vitro*	α-syn	Microglia	Fcγ receptor α-SYN NF-κB p65	FcγRs play an important role in the α-SYN interaction with microglia.	Inhibition of FcγRs
Yao et al. (2019)	BV2 microglia cells, human DA cell line, SH-SY5Y cells	–	*In vitro*	LPS MPTP	Microglia	p38 p62	The level of p38 and p62 elevates. They cause pro-inflammatory cytokines secretion	MicroRNA-124 have an effect on p38 and p62 negatively and on autophagy positively
Panicker et al. (2019)	HEK-293T cells	–	*In vitro*	AAV-αSyn	Microglia	Fyn kinase αSyn NLRP3-inflammasome CD36	β-Syn uptake in microglia is regulated by Fyn and CD36 α-syn activates NLRP3 inflammasome	Inhibition of the NLRP3 inflammasome and Fyn
Jo et al. (2019)	C57BL/6N wild-type (WT) mice	Male Age not specified	*In vivo*	MPTP		Gintonin, a ginseng-derived glycolipoprotein	Gintonin reduces the level of α-syn aggregation induced by MPTP in the SN and striatum of the mouse model	
Earls et al. (2019)	C57BL/6J mice	8-week-old males and females	*In vivo* Intrastriatal injection	PFF α-syn–injected	Microglia, Astrocytes, B, CD4+ T, CD8+ T, and natural killer cells	PFF α-syn	Intrastriatal injection of PFF α-syn leads to the activation of Microglia, Astrocytes, B, CD4+ T, CD8+ T, and natural killer cells in not only CNS but also peripheral lymphoid organs	
Hong et al. (2018)	C57BL/6 mice	Male Age not specified	*In vivo*	MPTP	Mast cells	TG2	Mast cells release TG2 after activation of NF-κB pathway by MPP+	
Neal et al. (2018)	C57 BL/6J mice	Sex and age not specified	*In vivo* Intraperitoneal injection	MPTP	Astrocyte	GPNMB	GPNMB activates the CD44 receptor leading to an anti-inflammatory phenotype in astrocyte	
Zhu et al. (2018)	Mouse primary astrocytes	–	*In vitro*	MPTP		Drd2 NLRP3 inflammasome	Dopamine D2 receptor limits NLRP3 inflammasome in astrocytes	
Ambrosi et al. (2017)	Sprague–Dawley rats	Male Age not specified	*In vivo* Unilateral infusion to the SN	6-OHDA	CD4+ T regulatory (Treg) cells		CD4+ T regulatory (Treg) cells are decreased in the circulation	
Kim B. W. et al. (2016)	C57BL/6 mice	8–12 weeks old Male	*In vivo* Intraperitoneal injections	MPTP	Microglia Astrocytes	LCN2	Expression of LCN2 increases in the nigrostriatal DA system as MPTP-induced PD in mice activates glia and astrocytes	Abrogation of LCN2 in the nigrostriatal DA system
Main et al. (2016)	C57Bl/6 wildtype mice and IFNAR1^−/−^ mice	Sex and age not specified	*In vivo*	MPTP		Type-1 IFNs	Type-1 IFNs contribute to neurodegeneration in PD	
Zhu et al. (2015)	Cell culture from ventral mesencephalic tissues of embryonic Sprague Dawley rats	–	*In vitro*	LPS	Astrocytes	IL-10	LPS treatment results in neural cell loss and activates astrocytes to release IL-10	IL-10
Shin et al. (2015)	Sprague Dawley (SD) rats, C57BL/6 mice	Sex and age not specified	*In vivo* Intranigral injection of pKr-2	Prothrombin kringle-2	Microglia	TLR4	Prothrombin kringle-2 (pkr-2) administration in rat and mouse brains increases TLR4 expression	Inhibition of pkr-2 induced increase of TLR4
Paumier et al. (2015)	Sprague Dawley rats	Three-month-old Male	*In vivo* Intrastriatal injection	α-syn PFF injection		α-syn	α-syn PFF injection leads to α-syn accumulation and neurodegeneration	
Li et al. (2013)	A53T human alpha-synuclein transgenic mice	Sex and age not specified	*In vivo*	A53T α-syn			A53T α-syn causes mitochondrial damage	
Kim et al. (2013)	SH-SY5Y human neuroblastoma, primary cortical neurons, rat and mouse primary microglia, BV2 murine microglial cell lines, and COS-7 cells Sprague-Dawley rats and C57BL/6 mice	Sex and age not specified	*In vitro/In vivo* Stereotaxically injection of AAV-αSyn to the putamen. Transportation of AAV-αsyn retrogradely to the substantia nigra.	α-syn	Microglia	TLR2 α-synuclein	α-syn is a ligand of TLR2 on microglia and causes a release of cytokines from the activated microglia	Drugs that affect TLR2 or extracellular α-syn
Gu et al. (2010)	A53T human alpha-synuclein transgenic mice	Sex and age not specified	*In vivo*	A53T α-syn	Astrocytes	COX-1 IL-1β	A53T α-syn expressed in astrocytes causes microglia activation and release of proinflammatory cytokines	
Fernagut et al. (2007)	Thy1-aSyn mice	10–12 weeks old Sex not specified	*In vivo*	Paraquat		α-syn	α-syn aggregates in the *substantia nigra* and is resistant to proteinase K	
Brochard et al. (2009)	C57BL/6J mice	Age not specified Male	*In vivo*	MPTP	CD4 + T cells		CD4 + T cell-dependent Fas/FasL cytotoxic pathway causes DA cell death	
Miklossy et al. (2006)	Rhesus monkeys (*Macaca mulatta*)	Sex and age not specified	*In vivo*	MPTP	Astrocyte	ICAM-1	ICAM-1 is upregulated in astrocytes which shows inflammation is significant in the pathogenesis of PD	Administration of anti-inflammatory agents
Giasson et al. (2002)	wild-type and A53T human alpha-synuclein expressing transgenic mice	Sex and age not specified	*In vivo*	A53T α-syn		α-syn	A53T α-syn causes neurodegeneration	

*TLR2, Toll-like receptor 2; GMF, glial maturation factor; CCL2, chemokine (C-C motif) ligand 2; UCP4, Uncoupling protein 4; LCN2, Lipocalin-2; mPGES-1, microsomal prostaglandin E synthase-1; PGE2, prostaglandin E2; HMGB1, High-mobility group box 1; TG2, Transglutaminase 2; PDE7, Phosphodiesterase 7; Drd2, dopamine D2 receptor; ICAM-1, intercellular adhesion molecule-1; COX-1, cyclooxygenase 1; IFN, Interferon*.

## Results

### Study Selection

**Figure 1:**
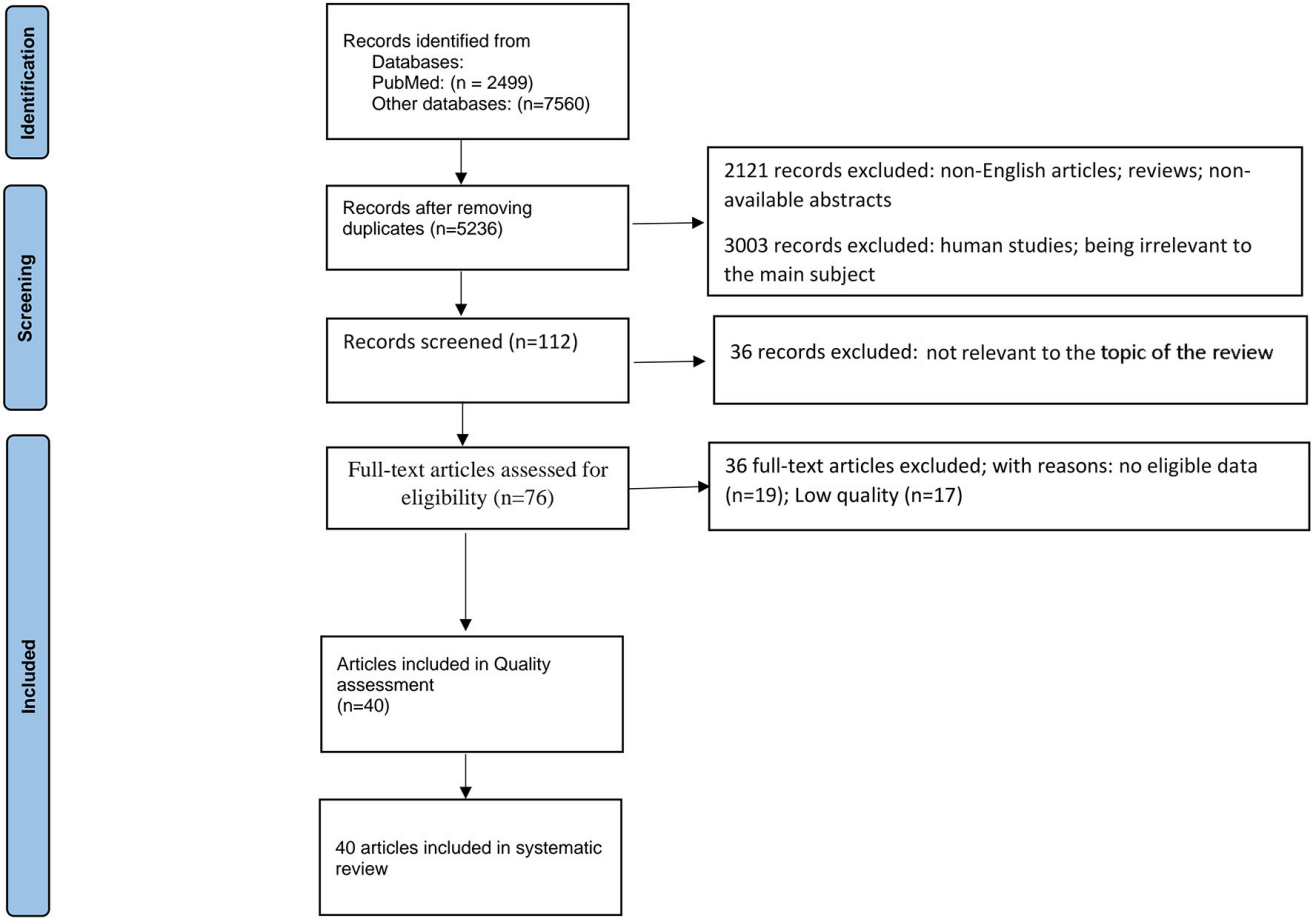
PRISMA 2020 flow diagram for systematic reviews which included searches of databases.

The current study has been run based on PRISMA checklist (Tore and Tuncel, 2009). At first, 2,499 papers were identified through the search on PubMed, and 7,560 were identified through searching other databases (Scopus, Google Scholar, Web of Science, and EMBASE). However, 4,823 of those 10,059 articles were excluded due to duplication in different databases. After screening the abstracts and titles of these obtained studies, 2,121 more papers were excluded due to unavailability of abstracts, or articles written in non-English, or being review articles. Moreover, 3,003 more papers were removed due to being irrelevant to the main subject or human studies. Finally, from the 112 remaining articles, 36 records excluded because of not relevant for the topic of the review. The full texts of the 76 remaining papers were fully assessed, and 36 more studies were excluded due to insufficient or unclear data (*n* = 19) and low quality (*n* =17). Finally, 40 articles, published before April 2022, were chosen for systematic review (Figure 1).

### Types of Studies Included at a Glance

The main results of all the papers included in this review are summarized first based on the neuroinflammatory pathways of PD. The method used to induce PD in animal models might be a key factor which affects the mechanism of neuroinflammation observed in a particular study; therefore, we reviewed the methods in both Table 1 and a separate subsection of the results. The other subsections are introduced based on the most frequently appearing topics and mechanisms discussed in the reviewed articles.

A total of 40 articles are included in this review. Twenty-one out of 40 studies used only *in vivo models*, 11 were based on *in vitro experiments*, and 8 studies employed both *in vitro* and *in vivo* experiments. A variety of methods were used in different studies to induce PD: 11 studies used MPTP, 10 α-Syn injection, 6 LPS, 5 A53T α-Syn transgenic mice, and 2 paraquat. Two studies used both 6-OHDA and LPS, 1 used both MPTP and LPS, 1 6-OHDA, and 1 used prothrombin kringle 2.

### Summary of Methods Used to Induce PD in Animal Models

Table 1 illustrates a variety of methods used by the reviewed studies to induce PD in animal models. Each method initiates its own pathway leading to PD-like pathology. Administration of MPTP to different animal species has been used extensively to model PD neuropathology. MPTP mimics the destruction of dopaminergic neurons of the substantia nigra pars compacta observed in PD. The mechanism by which MPTP causes damage to dopaminergic neurons involves a sequence of events including disturbance in the mitochondrial function, oxidative stress and respiratory failure. MPTP leads to inducible nitric oxide synthase (iNOS) overexpression. MPP+ is the active metabolite of MPTP which accumulates in dopaminergic (DA) neurons of the model after treatment with MPTP. MPP+ provokes the production of superoxide radicals which react with nitric oxide and generate peroxynitrite. This substance inhibits the function of many proteins including tyrosine hydroxylase. As a result, the production of dopamine is disturbed leading to damage of DA neurons (Przedborski et al., 2000; Burré et al., 2014, 2018). The loss of DA cells after MPTP administration has also been shown to activate microglia in the SN of rhesus monkeys (McGeer and McGeer, 2007). Therefore, *in vivo* and *in vitro* use of MPTP and its metabolite MPP+ can help elucidate the molecular mechanisms of the neuroinflammatory reactions in PD. Injection of lipopolysaccharide (LPS) and the following brain region-specific inflammation in mice provides *in vivo* models to investigate neurodegenerative diseases such as PD (Noh et al., 2014). LPS treatment in mice causes a decrease in the level of IL-4 and IL-10 but an elevation in the level of TNF-α, interleukin-1β, prostaglandin (PG) E_2_, and nitric oxide. IL-10 has a neuroprotective effect against LPS intoxication (Tansey and Goldberg, 2010). As shown in Table 1, some studies have used this method to create a PD model. 6-hydroxydopamine (6-OHDA) is another neurotoxin used to create PD models which affects nerve terminals as well as cell bodies and induces death of neurons through inhibition of the mitochondrial respiratory enzymes. In PD models 6-OHDA lesions are made in nigrostriatal dopaminergic pathways (Deumens et al., 2002). One of the studies used paraquat to induce PD *in vitro* (Trudler et al., 2021). Paraquat is a pesticide exposure to which is epidemiologically known to be a risk factor for PD (Berry et al., 2010). The rotenone-induced PD is another method, which is the best model to study mitochondrial complex I deficiency in PD (Greenamyre et al., 2003).

### α-Synuclein-Induced Neuroinflammation *in vivo* and *in vitro*

#### α-Synuclein: A Key Regulator of Glial Immune Responses

Synucleins are proteins highly expressed in the brain. There are three members in this protein family: α-, β-, and γ-synuclein. α-Synuclein is a pre-synaptic protein mostly found in nerve terminals (Goedert, 2001). Under physiological conditions, α-Syn interacts with many neuronal proteins to play a variety of functional roles such as inhibiting phospholipase D, regulating microtubules, elevating the rate of tau phosphorylation, etc. It also interacts with soluble NSF attachment protein receptors (SNAREs), which play a role in neurotransmitter release through meditating fusion of vesicles. This indicates that α-Syn is a likely contributor to the release of neurotransmitters (Burré et al., 2010, 2014, 2018). Point mutations in the gene encoding α-Syn (SNCA) are linked to the familial form of PD. A variety of mutations are known to be linked to PD including A53T, A30P, E46K, G51D, and H50Q. We will discuss some of these mutations in transgenic PD models in the following section in more details. Lewy bodies, the hallmark neuronal inclusions observed in neurodegenerative diseases including idiopathic PD, mainly consist of insoluble fibrillar α-syn protein (Goedert, 2001; Marques and Outeiro, 2012; Bendor et al., 2013). In pathological situations, soluble monomeric α-syn generates β-sheet-like oligomers (protofibrils), which form into amyloid fibrils. Amyloid fibrils, then, accumulate in Lewy bodies (Burré et al., 2015). Neurons can also release α-syn, inducing inflammatory responses of microglia (Kim et al., 2013). The fibrillar α-syn, but not its monomeric or oligomeric forms, is the main trigger of neuroinflammation in PD. It interacts with microglial TLR2 as well as another innate immune sensor, the nucleotide oligomerization domain-like receptor family, pyrin domain containing 3 (NLRP3), resulting in activation of the nuclear factor κB (NF-κB) and assembly of NLRP3 inflammasomes. This sequence of events ultimately leads to the release of TNF-α and IL-1β by the microglia, two potent pro-inflammatory cytokines that are known to play a role in the pathogenesis of PD (Gustot et al., 2015; Zhou et al., 2016; Panicker et al., 2019; Trudler et al., 2021). AMS-17 is known to inhibit NLRP3 pathways and activation of microglia. Further investigations using PD model mice indicate that microglial endocytosis of α-syn, impairment of lysosomal functions, and the release of lysosomal protease cathepsin B into the cytoplasm are required for α-syn-induced assembly of the NLRP3 inflammasomes in microglia. α-Syn also negatively regulates the AMP-activated protein kinase (AMPK)-mediated autophagy, which leads to the intracellular accumulation of reactive oxygen species *(*ROS), followed by activation of the NLRP3 inflammasome (Zhou et al., 2016). Furthermore, α-syn binds to the microglial CD36 receptor, resulting in the activation of Fyn kinase and subsequently the activation of the NF-kB pathway. Fyn kinase also participates in the activation of NLRP3 since it is decreased in the fyn^−/−^ mice injected with adeno-associated virus overexpressing α-syn (AAV-αSyn) compared to the wild-type animals (Panicker et al., 2019). In addition, Fcγ receptors (FcγR) located on the surface of microglia can mediate α-syn intracellular trafficking leading to pro-inflammatory activation of these cells (Cao et al., 2012). α-Syn can also promote neuroinflammation by interacting with astrocytes since it has been shown to upregulate secretion of interleukin IL-6 and expression of intercellular adhesion molecule-1 (ICAM-1) by human astrocytes. Notably, the PD-causing mutations of α-syn upregulate its potency as a pro-inflammatory stimulant of astrocytes (Klegeris et al., 2006; Jo et al., 2019). In addition to stimulating glial cells, α-syn facilitates aggregation of other inflammatory proteins. For example, co-localization and co-aggregation of α-syn with S100A9 protein have been observed in Lewy bodies and neuronal cells in the SN and frontal lobe areas of PD patients. S100A9 is a member of a family of structurally homologous calcium-binding S100 proteins, which are involved in many inflammatory and neurodegenerative diseases (Srikrishna, 2012; Markowitz and Carson, 2013; Horvath et al., 2018). Aggregated a-syn leads to downregulation of progranulin (GRN) gene in microglia which affects immune functions of these cells (Sarkar et al., 2020a). Another effect of α-syn is related to metabotropic glutamate receptor 5 (mGluR5). This receptor has a neuroprotective role, but its lysosome-dependent degradation occurs faster as a result of synucleinopathy (Zhang et al., 2021). Even though α-syn has been demonstrated to be an initiator in neuroinflammation processes, some studies indicated that inflammation can occur before synucleinopathy (Lai et al., 2021).

#### α-Syn and Transgenic PD Mouse Models

To further study the pathophysiology of α-syn, various transgenic mouse models of PD have been created including α-syn knockout models and models overexpressing wildtype or mutated human α-syn (Fernagut and Chesselet, 2004). α-Syn KO mice are used to identify the role of α-syn in PD pathogenesis. A study on α-syn KO mice demonstrated that these PD models are resistant to acute administration of MPTP, which highlights the key role of α-syn in increasing the vulnerability of DA neurons to neurodegeneration when exposed to environmental neurotoxins (Dauer et al., 2002). As previously mentioned, two missense mutations of α-syn gene are linked to PD: A53T and A30P. Homozygous transgenic A30P^*^A53T α-syn mice manifest many features of PD phenotype and are useful models to study this disease (Kilpeläinen et al., 2019). Mutant α-syn in transgenic A53T mice does not form aggregates but is distributed in different parts of neurons abnormally leading to motor impairment in transgenic mice followed by further paralysis and death (Giasson et al., 2002; Gispert et al., 2003). In transgenic mice which overexpressed A53T α-syn selectively in astrocytes, microglia became reactive and produced proinflammatory cytokines, such as IL-1β, and upregulated cyclooxygenase (COX)-1 leading to neurodegeneration (Gu et al., 2010). Accumulation of A53T α-syn in transgenic mice leads to peripheral inflammation and motor deficits (La Vitola et al., 2021). A53T α-syn has a destructive effect on mitochondrial function in the DA neurons of transgenic mice. It causes damage to mitochondrial transport and respiratory mechanisms (Li et al., 2013). Some other transgenic mouse models are also created to investigate PD such as Thy1-aSyn and α-syn pre-formed fibril (PFF)-injected models. Intrastriatal injection of PFF α-syn leads to the activation of microglia, astrocytes, B, CD4+ T, CD8+ T, and natural killer cells in not only CNS but also peripheral lymphoid organs (Earls et al., 2019). Thy1-aSyn models overexpress human wildtype α-syn by the murine Thy-1 promoter. In this model, many manifestations of sporadic PD are seen including inflammation, biochemical and molecular changes resembling those observed in PD (Chesselet et al., 2012). To study pre-clinical stages of PD, Thy1-aSyn transgenic mice are helpful as in this model high levels of α-syn cause no DA neuronal death up to 8 months (Fleming et al., 2008).

### Role of NF-κB Pathway in Neuroinflammation

As described above, the interaction of α-syn with microglia causes activation of NF-kB, which is central to a broad range of neuroinflammatory processes (Tobon-Velasco et al., 2014); therefore, inhibition of this signaling pathway could be a therapeutic target for PD. Panicker et al. treated Fyn^−/−^ mice with LPS to evaluate the role of Fyn in NF-κB activation (Greenamyre et al., 2003). NF-kB signaling in microglia can be activated by the DAMPs released from damaged CNS cells, such as HMGB1, IL-33, ATP, cytochrome C, mitochondrial DNA, and several different heat shock proteins (HSP) (Klegeris, 2021). HMGB1 is a prototypical DAMP present in most nucleated cells, which can be actively secreted or passively released from stimulated and necrotic cells, respectively. a-Syn-induced upregulation of CXCL12 and its release from microglia through TLR4/IκB-α/NF-κB pathway results in microglia migration to the SN (Li Y. et al., 2019). GMF causes NLRP3 inflammasome inhibition and decreases levels of IL-1β and IL-18 (Javed et al., 2020). An *in vitro* study showed that knockdown of HMGB1 alleviated upregulation of NF-κB signaling and inflammatory responses; therefore, anti-HMGB1 monoclonal antibody therapy should be considered as a potential treatment strategy for PD (Nishibori et al., 2019; Huang et al., 2020). The pro-neuroinflammatory effects of DAMPs in the CNS can be counterbalanced by several different resolution-associated molecular patterns, including HSP10, αB-crystallin, prothymosin α, and binding immunoglobulin protein (BiP), also known as HSP70 (Wenzel et al., 2020). Thus, an *in vitro* model of PD was used to demonstrate that HSP70 inhibited the mRNA and protein expressions of NF-κB along with another key pro-inflammatory signaling molecule signal transducer and activator of transcription (STAT)-3 (Li et al., 2019). Notably, select miRNAs, such as miR-124 and miR-7, can also inhibit the progression of neuroinflammation in PD models by modulating NF-κB signaling pathways (Zhou et al., 2016; Yao et al., 2019). One study showed the Lipoic acid (LA) has an anti-inflammatory effect by inhibiting the p53/NF-κB pathway in an LPS-induced model of PD (Mao et al., 2021).

### Contribution of Mast Cells to Neuroinflammation in PD Models

The essential role of mast cells in neuroinflammation is supported by several studies. Kempuraj et al. showed that exposure of murine and human mast cells to MPP+ induced release of chemokine c-c motif ligand 2 (CCL2), which in turn has been implicated in the pathogenesis of PD (Kempuraj et al., 2016). MMP+ treatment-induced secretion of IL-33 and a high level of ROS generation by murine mast cells. In addition, mouse mast cell protease (MMCP)-6 and MMCP-7 triggered the release of IL-33 from glia-neuron mixed cultures and primary mouse astrocytes. All these events involved the activation of the NF-kB pathway demonstrating its critical role in neuroinflammation associated with PD (Kempuraj et al., 2019). Furthermore, MPP+ activated NF-κB in mast cells leading to upregulation of transglutaminase 2 (TG2) and subsequent release of the pro-inflammatory TNF-α and IL-1β. This study also demonstrated that TG2-expressing mast cells recruited into SN tissues might contribute to neuroinflammation in PD (Hong et al., 2018).

### Role of Adaptive Immunity in PD Models

Nigrostriatal damage correlates with complex alterations in both central and peripheral immunity (Ambrosi et al., 2017). Regarding *in vivo* studies, some immunological features change in MPTP-treated mice as a result of the damage to central dopaminergic cells (Bieganowska et al., 1993). In 6-OHDA-treated rats, a decrease in the percentage of circulating CD4+ T regulatory (Treg) cells was observed (Ambrosi et al., 2017). Upregulation of a-syn provokes adaptive immune system (Theodore et al., 2008). Upregulation of the major histocompatibility complex II (MHCII) protein in CNS myeloid cells and recruitment of CD4+ and CD8+ T cells into the CNS occur due to α-syn accumulation (Williams et al., 2021). One study demonstrated that CD3+/CD4+ T cells are recruited into the perivascular parenchyma of the neocortex, hippocampus, and striatum in α-syn transgenic mice. This study supports the role of adaptive immune cells in neuroinflammation in synucleinopathies (Iba et al., 2020). Degeneration of DA cells occurs through a CD4+ T cell-dependent Fas/FasL cytotoxic pathway (Brochard et al., 2009). CD4+ and CD8+ T cells, in an α-syn rat model of PD, cause an upregulation of MHCII in microglia and significant loss of DA neurons (Subbarayan et al., 2020). One study on A53T α-syn mice indicated that T cells contribute to the neurodegeneration caused by α-syn, while B cells have no neuroprotective effect against this neurodegeneration (Karikari et al., 2022). Taken together, these studies indicate that the adaptive immune system is associated with neuroinflammation and neurodegeneration in PD.

### Other Factors Contributing to Neuroinflammation in PD Models

TLR4 is a cell-surface protein that interacts with bacterial endotoxin and other pathogen-associated molecular patterns (PAMPs) as well as several different DAMPs (Leitner et al., 2019). This receptor is expressed by many different cell types including glial cells. TLR4 activation causes motor impairment in MPTP-treated mice. TLR4 signaling has been suggested to initiate neuroinflammation in PD since microglia express this receptor and α-syn activates it. A decrease in the inflammatory response to α-syn oligomers is observed in murine macrophages derived from bone marrow. In TLR4-deficient mice, the activation of microglia and astrocytes is inhibited by the suppression of NF-κB and the NLRP3 inflammasome signaling pathways (Campolo et al., 2019; Hughes et al., 2019; Shao et al., 2019). An elevated level of microglial TLR4 damages the nigrostriatal DA system. The increase in this receptor occurs after the administration of prothrombin kringle-2 (pKr-2) in rat and mouse brains, which indicates pKr-2 as a new potential TLR4-linked therapeutic target for PD (Shin et al., 2015). Lee et al. report that apoptosis signal-regulating kinase 1 (ASK1) is responsible for the MPTP-induced glial activation and neurotoxicity (Lee et al., 2012). PD model mice were also used to identify glial lipocalin-2 (Lcn-2) as a protein contributing to the disease pathogenesis since its levels were increased in the SN and striatum of the MPTP-treated animals (Kim B. W. et al., 2016).

A microsomal isoform of prostaglandin E synthase-1 (mPGES-1) plays a vital role in the inflammatory processes of peripheral tissues and the CNS by producing the inflammatory PGE2. It contributes to clinical manifestations of inflammation, such as pyrexia and pain, and has been shown to contribute to accelerated neuronal death in PD (Uematsu et al., 2002; Engblom et al., 2003; Kamei et al., 2004). Furthermore, mPGES-1 is upregulated in nigrostriatal DA neurons from postmortem PD brain specimens and the 6*-*hydroxydopamine (6-OHDA) model of PD. Elevated PGE2 produced by this enzyme is believed to contribute to the DA neuronal death in this model (Ikeda-Matsuo et al., 2019). The type 7 cyclic nucleotide phosphodiesterase 7 (PDE7) is another pro-inflammatory enzyme revealed by several pre-clinical models to contribute to PD pathogenesis. PDE7 inhibitors display anti-inflammatory and neuroprotective activities and can stimulate adult neurogenesis both *in vivo* and *in vitro* (Morales-Garcia et al., 2011, 2015, 2017). Recently, small non-coding RNAs, such as microRNAs (miRNAs), have been identified as biomarkers of neurodegenerative processes and shown to be involved in PD pathogenesis (Kim et al., 2016). Specific miRNAs bind to the three prime untranslated regions (3′-UTRs) of target mRNAs regulating gene expression in the post-transcriptional phase (Bartel, 2004). In the MPTP model of PD, type-I IFNs mediate neuroinflammation in its early stages and worsen PD pathology (Main et al., 2016).

### Comparison Between Different Cell Lines Used to Model PD *in vitro*

As seen in Table 1, BV2 murine microglial cells (four articles) and and SH-SY5Y human neuroblastoma cell line (four articles) are the two most commonly-used cell lines to induce PD *in vitro* in the articles included in this review. Six *in vitro* studies used rat/mice primary neuron/glia to model PD rather the previously mentioned cell lines. Primary neuronal cultures are useful PD models obtained from the embryonic rodent brain which resemble the morphology and physiology of human neurons (Lopes et al., 2017a). Experimental variations and difficulty in maintenance are some disadvantages of this model (Slanzi et al., 2020). To study the role of microglial cells in PD pathology, the immortalized murine microglial cell line BV-2 has been widely used instead of primary microglial cells. LPS administration in this model leads to pathological pattern of PD similar to that observed in microglial cells *in vitro* and *in vivo* (Henn et al., 2009). SH-SY5Y human neuroblastoma cells is a widely-used PD model which mimics some aspects of DA neuron phenotype, such as the expression of tyrosine hydroxylase, dopamine-β-hydroxylase, and dopamine transporter (Hong-rong et al., 2010). SH-SY5Y neuroblastoma can be differentiated to cells that are similar to cholinergic, dopaminergic, or noradrenergic neurons (Slanzi et al., 2020). However, one disadvantage of this model is that there is no standard differentiation protocol and variation in the source of the cells and their culture maintenance techniques lead to different results. In order to provide a PD model, SH-SY5Y neuroblastoma lineage is manipulated both chemically and genetically. MPP+, 6-OHDA, and rotenone treatment as well as overexpression of mutated α-syn are some examples. SH-SY5Y cells cannot transform MPTP to its metabolite and must be treated by MPP+ itself (Xicoy et al., 2017). Some other cell lines are also mentioned in Table 1. For example, PC12 cells are derived from rat pheochromocytoma of the adrenal medulla. These cells produce and secrete catecholamines. However, due to their tumoral origin, they may manifest altered signaling pathways (Slanzi et al., 2020). HEK 293 cell line (or immortalized human embryonic kidney cells) has been used for large-scale experiments but their non-neuronal origin is the main disadvantage of this model (Falkenburger and Schulz, 2006). Animal models of PD are appropriate for studies on motor deficits in PD as the disease progresses. However, since PD is a human-specific neurodegenerative diseases, its pathology, such as damage to DA neurons, needs to be induced. No animal model has been able to recreate all the PD aspects so far. *In vitro* models provide a controlled environment to investigate the disease mechanisms. However, these models do not have the complexity of CNS. In addition, the source of cells used, their morphology, physiology, and maintenance under different culture conditions result in different outcomes (Lopes et al., 2017a).

## Discussion

The complex pathological features of PD include the death of dopaminergic neurons in SN as well as α-syn aggregation accompanied by neuroinflammation (Hirsch and Hunot, 2009; Tansey and Goldberg, 2010; Hirsch et al., 2012; Guo et al., 2017; Mohamadkhani, 2018). In the current systematic review, we considered 40 original *in vitro* and *in vivo* studies describing the molecular mechanism, signaling pathways, and other factors that potentially contribute to the PD pathophysiology in animal models. Our review highlights the following main findings: (1) the methods applied to create PD animal models include treatment of the animals with MPTP, LPS, 6-OHDA, paraquat, and rotenone, in addition to transgenic animal models (Giasson et al., 2002; Miklossy et al., 2006; Fernagut et al., 2007; Gu et al., 2010; Li et al., 2013; Paumier et al., 2015; Zhu et al., 2015, 2018; Kempuraj et al., 2016, 2019; Kim B. W. et al., 2016; Main et al., 2016; Hong et al., 2018; Neal et al., 2018; Earls et al., 2019; Ikeda-Matsuo et al., 2019; Jo et al., 2019; Li Y. et al., 2019; Panicker et al., 2019; Yao et al., 2019; Huang et al., 2020; Javed et al., 2020; Morales-Garcia et al., 2020; Sarkar et al., 2020a; Lai et al., 2021; La Vitola et al., 2021; Trudler et al., 2021; Williams et al., 2021; Zhang et al., 2021). (2) α-syn is an important regulator of glial immune responses, which can induce neuroinflammation and, thus, lead to PD development (Cao et al., 2012; Kim et al., 2013; Ikeda-Matsuo et al., 2019; Jo et al., 2019; Panicker et al., 2019); (3) the NF-κB pathway, induced by α-syn, contributes to the neuroinflammatory process during PD progression (Zhou et al., 2016; Li et al., 2019; Nishibori et al., 2019; Yao et al., 2019; Huang et al., 2020; Wenzel et al., 2020; Klegeris, 2021); (4) mast cells are important contributors to the disease pathogenesis in PD mouse models (Kempuraj et al., 2016, 2019; Hong et al., 2018); (5) adaptive immune system plays role in neuroinflammation and neurodegeneration in PD pathogenesis (Bieganowska et al., 1993; Brochard et al., 2009; Ambrosi et al., 2017; Iba et al., 2020). (6) additional factors contributing to PD neuroinflammation involve the pKr-2 protein, which can facilitate neuroinflammation and PD progression in rodent models by upregulating microglial TLR4 (Shin et al., 2015); mPGES-1, which is upregulated in the SN of 6-OHDA-induced PD models and could lead to neurotoxicity (Uematsu et al., 2002; Engblom et al., 2003; Kamei et al., 2004; Morales-Garcia et al., 2011, 2015, 2017; Kim et al., 2016; Ikeda-Matsuo et al., 2019); and type-I IFN, which aggravates PD by inducing neuroinflammation in the early stages of this disease (Main et al., 2016).

**Figure 2:**
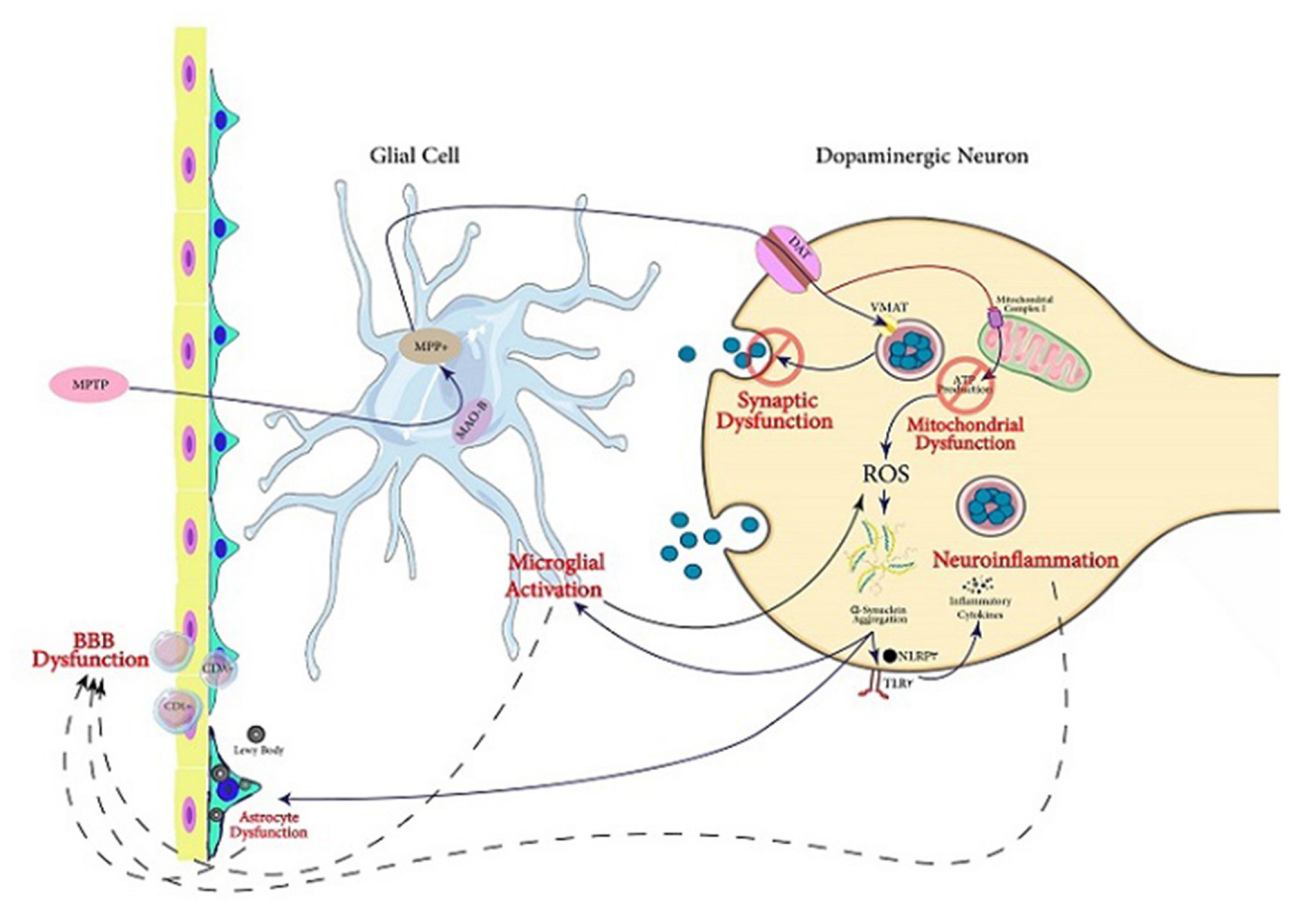
Servier (2022) mechanisms involved in PD pathogenesis. In this figure, the mechanisms following MPTP exposure through which dopaminergic cell death occurs are illustrated. The active metabolite of MPTP, MPP^+^, is produced in glial cells and transfers into dopaminergic neurons *via* DA transporter. In the neuron, this activated metabolite causes synaptic dysfunction as well as mitochondrial dysfunction which triggers aggregation of α-Syn. It also can lead to neuroinflammation and microglial activation. Acting together, the mentioned mechanisms can lead to dysfunction of BBB, which is one of the main pathological findings in PD, in addition to Lewy bodies and α-Syn aggregation.

MPTP is one of the agents used to create animal Parkinson models (Miklossy et al., 2006; Kim B. W. et al., 2016; Main et al., 2016; Hong et al., 2018; Neal et al., 2018; Zhu et al., 2018; Kempuraj et al., 2019; Yao et al., 2019). For performing its toxicity, MPTP is initially transformed to MPP^+^. In DA neurons, ASK1 conveys the MPP^+^-induced signals leading to the generation of glial-activating molecules like COX-2. During the MPTP-induced toxicity in mice, ASK1 signaling plays a significant role as a connection between neuroinflammation and oxidative stress (Lee et al., 2012). A review by Guo et al. illustrates that ASK1 signaling is linked to the pathogenesis of several neurodegenerative diseases including PD (Guo et al., 2017). Therefore, ASK1 is important in the neurotoxicity caused by MPTP treatment. Furthermore, it is suggested that ASK1 may be a target for treating or preventing PD and other neurodegenerative diseases (Guo et al., 2017). Treatment of mice with MPTP also increases glial Lcn-2 levels, according to Kim B. W. et al. (2016). This is consistent with previously reported inflammatory functions of this protein potentially playing a role in various age-related CNS diseases. Lcn-2 has been shown to promote cell death and iron dysregulation, in addition to neuroinflammation, leading to cognitive impairments. Therefore, elevated Lcn-2 levels can be considered a risk factor for age-related CNS disorders (Dekens et al., 2021). Additionally, it is indicated that MPP+ causes superoxide radicals to form, which combine with nitric oxide to form peroxynitrite. Many proteins, including tyrosine hydroxylase, are inhibited by this chemical. As a result, dopamine synthesis is disrupted, leading to injury of DA neurons (Liberatore et al., 1999; Przedborski et al., 2000, 2004). McGeer et al. demonstrated that in the SN of rhesus monkeys, the loss of DA cells after MPTP treatment caused microglia activation, which contributes to neuroinflammation (McGeer and McGeer, 2007) (Figure 2).

An LPS-induced mouse model of neuroinflammation has been shown to be a useful tool for studying the pathogenic mechanisms behind neurodegeneration and testing possible therapeutic agents (Noh et al., 2014; Zhu et al., 2015; Panicker et al., 2019; Yao et al., 2019; Morales-Garcia et al., 2020; La Vitola et al., 2021; Zhang et al., 2021). The TLR4 and NF-κB signaling pathway is activated by LPS injections, which stimulates microglia. This causes release of IL-6, TNF-α, and insulin-like growth factor 1 (IGF-1), which can be beneficial or harmful to surrounding tissues depending on conditions (Wyss-Coray and Mucke, 2002; Block et al., 2007; Noh et al., 2014). In both the mouse and the rat, LPS injection causes the expression of pro-inflammatory markers such as TNF-α and IL-1b in the entire brain and plasma (Qin et al., 2007; Henry et al., 2009; Nikodemova and Watters, 2011; Oskvig et al., 2012; Molteni et al., 2013). LPS injections produce systemic inflammation and neuroinflammation, which lead to an increase in Aβ levels and neuronal cell death, resulting in cognitive impairment. Thus, systemic inflammation can play a role in the progression of cognitive impairments observed in Alzheimer's disease (AD) and PD (Zhao et al., 2019). According to the study by Morales-Garcia et al. (2020), PDE7 is involved in the progression of neuronal damage in neurodegenerative illnesses, in part due to its role in the regulation of neuroinflammation, suggesting that it could be a key factor in the advancement of PD. In fact, this study detected a strong rise of PDE7 expression, both *in vitro* and *in vivo*, primarily in microglial cells, implying that PDE7 expression is critical for the neuroinflammatory response triggered by these cells, which leads to an increase in DA neuron degeneration (Morales-Garcia et al., 2020).

**Figure 3:**
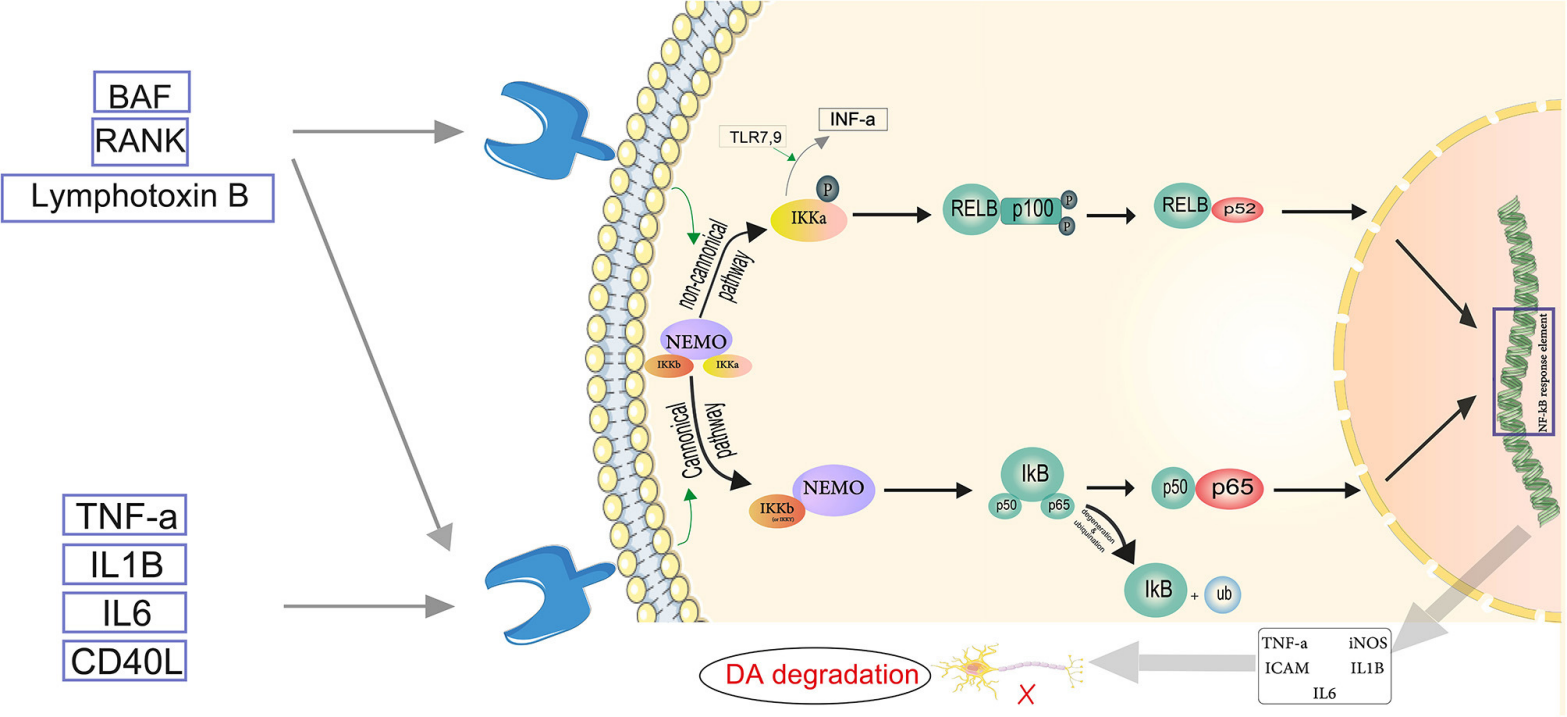
Servier (2022) the neuroinflammatory cascade mediated by NF-κB. TNF-α, IL-1β, IL-6, and CD-40L are proinflammatory substances activating the canonical pathway. In the canonical pathway, an inhibitor of κβ kinase (IKK) β (or IKKγ) is required for NF-κB activation. IKKβ phosphorylates Iκβ. The regulatory subunit of the IKK complex is the NF-κB essential modulator (NEMO). In the cytosol, IκB is degraded by proteasomes, and the phosphorylated heterodimer of NF-κB (p50–p65) is transferred to the nucleus and binds to the NF-κB response element. Thus, pro-inflammatory mediators such as TNF-α, IL-1β, IL-6, iNOS, and ICAM become activated, which play role in the degradation of dopaminergic neurons (DA). In the non-canonical pathway, NEMO phosphorylates IKK-α and induces proteasomal destruction as well as proteasomal processing of p100, a subunit of the NF-B heterodimer, creating the p52-RELB active heterodimer. IKκ induces INF-α production, triggered by TLR7,9. The p52-RELB active heterodimer enters the nucleus and binds to the NF-κB response element, regulating the expression of pro-inflammatory factors. Eventually NF-κB mediated neuroinflammation plays role in Parkinson's disease through DA degradation.

Another agent used for creating PD animal models is 6-OHDA (Ikeda-Matsuo et al., 2019; Morales-Garcia et al., 2020). 6-OHDA was the first chemical agent shown to have selective neurotoxic effects on catecholaminergic pathways (Ungerstedt, 1968; Sachs and Jonsson, 1975). 6-OHDA causes catecholaminergic neuron degeneration by using the same catecholamine transport mechanism as dopamine and norepinephrine (Figure 3) in the substantia nigra, the nigrostriatal tract, or the striatum to specifically target the nigrostriatal DA system (Perese et al., 1989; Przedbroski et al., 1995). DA neurons start degenerating 24 h after 6-OHDA (Przedbroski et al., 1995; Schwarting and Huston, 1996) injections into the SN or the nigrostriatal tract, and striatal dopamine is reduced 2–3 days later (Faull and Laverty, 1969). There is substantial evidence that oxidative stress plays a role in the neurotoxic effects of 6-OHDA. In the presence of iron, 6-OHDA-induced degeneration has been linked to the production of hydrogen peroxide and hydroxyl radicals (Sachs and Jonsson, 1975). The fact that intranigral iron injection has similar neurotoxic effects to 6-OHDA suggests that iron may play a role in 6-OHDA-induced degeneration. Furthermore, investigations have shown that 6-OHDA causes a decrease in glutathione peroxidase (GSH) and superoxide dismutase (SOD) activity as well as an increase in malondialdehyde levels in the striatum (Perumal et al., 1992; Kumar et al., 1995). 6-OHDA is similarly harmful to mitochondrial complex I, resulting in the generation of superoxide free radicals (Hasegawa et al., 1990; Cleeter et al., 1992).

Paraquat, a well-studied neurotoxic agent, is commonly regarded as one of the environmental elements contributing to PD (Fernagut et al., 2007; Huang et al., 2020). Because of its structural similarities to MPP+, Paraquat has presented as a possible risk factor for PD. Paraquat can pass the blood-brain-barrier, but only to a limited degree. It induces a dose-dependent decrease in DA nigral neurons and striatal DA innervation, and subsequent reduced mobility, when administered systemically to mice (Boireau et al., 1995). Paraquat method of action is thought to entail oxidative stress, and its harmful effects could be mediated through the mitochondria because of its structural resemblance to MPP+ (Betarbet et al., 2002). Paraquat has been shown in several studies to cause neuroinflammation and microglial activation. Underlying inflammatory processes greatly increase the sensitivity of DA neurons to toxic damage (Purisai et al., 2007; Mitra et al., 2011). According to a study by Ishola et al. TNF-α levels in the midbrain were considerably elevated by Paraquat, indicating neuroinflammation (Ishola et al., 2018). TNF-α is a cytokine that makes up the acute phase reaction and is a cell signaling protein implicated in inflammatory cascades (Tweedie et al., 2007; Ishola et al., 2013). Xiao et al. reported that Paraquat could activate BV-2 microglia and cause neuroinflammation. This involves inhibition of Akt1 activation, mediated by the increased ROS. Also, Paraquat treatment was followed by a significant increase in the expression of M1 microglia markers including TNF-α, IL-1β, and IL-6. Therefore, Paraquat elevated the M1 phenotype of BV-2 microglia (Xiao et al., 2022). According to Mitra et al. Paraquat induced ROS production and differential α-syn expression, which promoted neuroinflammation. This was characterized by area-specific changes in microglial cell localization and appearance, as well as an increase in TNF-α expression patterns in the substantia nigra, frontal cortex, and hippocampus (Mitra et al., 2011). Paraquat may also act through HGMB1 (Huang et al., 2020). In SH-SY5Y cells, a well-established *in vitro* model for PD research, Paraquat exposure resulted in a significant increase in HMGB1, which was translocated to cytosol and then released into the extracellular milieu of SH-SY5Y cells in a concentration and time-dependent manner. The activation of the RAGE-P38-NF-kB signaling pathway and the generation of inflammatory cytokines such as TNF-α and IL-6 were both reduced when HMGB1 was knocked out. These findings suggest that HMGB1 plays role in Paraquat-induced cell death by increasing neuroinflammatory responses and activating RAGE signaling pathways (Huang et al., 2020).

Another agent used for inducing PD in animal models is rotenone. This agent is an inhibitor of mitochondrial complex I, which is neurotoxic to non-DA and DA neurons (Chen et al., 2006; Choi et al., 2015). This is to some extent because of its inhibitory impact on mitochondria and also causing elevation of oxidative stress. It is indicated in studies using primary cultured glia or microglia that rotenone triggers oxidative stress and neuroinflammation, leading to elevated secretion of pro-inflammatory cytokines (Ye et al., 2016). According to Main et al. type-I IFNs play critical role in mediating the neuroinflammation caused by rotenone in both primary cultured glia and neurons in *vitro*. They also observed a neuroprotective effect by attenuating type-I IFNs signaling. This confirms the important role of these cytokines in neuroinflammation, which leads to death of neurons in chronic neuropathologies (Main et al., 2017) as was shown in another study by Main et al. on MPTP-induced mice (Main et al., 2016).

According to this systematic review, α-syn has a potentially significant role in the pathogenesis of PD. α-Syn performs many functions through its interactions with different proteins (Burré et al., 2018). It can inhibit phospholipase D (PLD). Many experimental studies, clearly show that α-syn and PLD have a functional interaction. PLD2 overexpression in the rat substantia nigra pars compacta, for example, induced dopaminergic neuron death due to increased lipase activity, while α-syn co-expression decreased PLD2 toxicity (Mendez-Gomez et al., 2018). Furthermore, PLD1 controls autophagic flux and clearance of α-syn aggregates (Bae et al., 2014), whereas overexpression of wild-type α-syn in human neuroblastoma cells reduces PLD1 expression (Conde et al., 2018). An important interaction of α-syn is with SNARE, which facilitates the formation of synaptic vesicles (Burré et al., 2010, 2014, 2018; Huang et al., 2019). SNARE complex assembly requires monomeric α-syn, and deficiencies in this protein can impair vesicle formation. Aggregated α-syn, on the other hand, prevents SNARE-mediated membrane fusion (Hawk et al., 2019). The findings explain a potential mechanism for SNARE-mediated neuronal dopamine release deficiencies leading to neurodegeneration due to a lack of monomeric α-syn and/or increased insoluble α-syn aggregation. However, since SNARE proteins are required for a variety of membrane fusion processes, changes in α-syn could affect vesicle production in a variety of cell types, particularly during fetal development when α-syn is most widely expressed (Baltic et al., 2004). Because vesicle formation is essential for microglia to phagocytose and transfer extracellular cargo to the lysosome for destruction, the deficiency of endogenous α-syn may have an impact on vesicle formation. During the beginning phase of autophagy, SNARE proteins are necessary for the synthesis of precursor vesicles that convert to the phagophore (Wang et al., 2020). This microglial process aids in the elimination of harmful proteins and protects against neuron-derived α-syn aggregation (Choi et al., 2016). Microglia's ability to remove misfolded α-syn could be harmed if vesicle production during autophagy is impaired and consequently contribute to aggregation of α-syn. The SNARE complex is also involved in vesicle exocytosis during microglia cytokine release (Murray et al., 2005). One probable reason for decreased cytokine release is α-syn interaction with SNAP23, a subunit of SNARE complex. Many microglial activities rely on vesicle production to protect against harmful protein buildup and to enhance inflammatory responses in the brain (Gardai et al., 2013).

α-sSyn induces neuroinflammation directly. This process is initiated by α-syn activating microglial TLR2 and NLRP3, which ultimately increases their secretion of IL-1β, TNF-α, and other pro-inflammatory cytokines (Panicker et al., 2019). For example, Bauernfeind et al. (2009) and Qiao et al. (2012) have demonstrated that α-syn is recognized by microglial TLR2, leading to activation of the NF-κB pathway and subsequent production of IL-1β (Bauernfeind et al., 2009; Qiao et al., 2012). Wang et al. (2019) highlight that NLRP3 inflammasome activation not only induces IL-1β secretion by microglia, but also causes a type of inflammatory cell death known as pyroptosis leading to rupture of microglial plasma membrane and further release of IL-1β (Wang et al., 2019). A meta-analysis by Qin et al. showed that peripheral levels of several inflammatory cytokines including TNF-α and IL-1β are higher in PD patients compared to healthy controls (Qin et al., 2016). IL-1β released from microglia has been suggested to participate in inflammatory responses that cause impairment of DA neurons (Block et al., 2007). Injection of IL-1β into the SN of rats induces the death of DA neurons, which is the pathological feature of PD (Ferrari et al., 2006; Block et al., 2007). This, in turn, increases the release of α-syn and creates a vicious circle that amplifies neuroinflammation and accelerates the pathogenesis of PD. Due to its critical role in neuroinflammation, inhibition of NLRP3 pathways is a recently suggested therapeutic strategy for PD (Wang et al., 2019). FcγR on the surface of microglia can mediate α-syn intracellular trafficking, causing microglia to become pro-inflammatory (Cao et al., 2012). According to a study by Javed et al. GMF inhibits the NLRP3 inflammasome and leads to a decrease in the levels of IL-1β and IL-18 (Javed et al., 2020). Miklossy et al. showed that ICAM-1 is upregulated in astrocytes of PD and MPTP-treated monkeys. Also, lymphocyte function-associated antigen 1 (LFA-1) was increased in the reactive microglia. Therefore, inflammation is a potential factor in PD pathogenesis (Miklossy et al., 2006).

The NF-κB pathway is found to be a significant mechanism driving neuroinflammatory reactions (Kempuraj et al., 2019). The two major routes involved in the activation of NF-κB are the canonical or classical pathway and the non-canonical or alternate pathway. The canonical pathway involves dimers of Rel proteins p50 and p65 forming complexes with inhibitory complex IκBα in the cytosol, where they are activated and regulate the production of pro-inflammatory cytokines (Lawrence, 2009). TNF, LPS, IL-1β, and T cell receptor or B cell receptor, as well as other cell, surfaces receptors such as TLRs, TNF receptor, and IL-1 receptor, activate NF-κB throughout the canonical pathway (Baeuerle and Baltimore, 1996). Members of the TNF receptor superfamily, such as B cell-activating factor (BAF), receptor activator of NF-κB (RANK), lymphotoxin B (LT) receptor, and CD40, activate the non-canonical NF-κB pathway in response to diverse stimuli. These receptors also activate the canonical pathway at the same time. Only IKKα homodimers, not IKKβ or IKKγ implicated in the canonical pathway for IκB phosphorylation, are responsible for non-canonical NF-κB pathway activation (Singh et al., 2020) (Figure 3). Inhibition or capture of HMBG1 can suppress this pathway and has been considered as a therapeutic approach. A review by Nishibori et al. illustrates that administration of anti-HMGB1 monoclonal antibodies inhibits Dneuronal loss in a 6-OHDA rat model of PD by suppressing ROS production and neuroinflammation (Nishibori et al., 2019). The protective effects of miR-124 and miR-7 against inflammation may make them protective in PD as already suggested by Titze-de-Almeida and Titze-de-Almeida, who describe the potential benefits of miR-7 replacement therapy in this disease (Titze-de-Almeida and Titze-de-Almeida, 2018). Microglia migration to the substantia nigra is triggered by α-syn-induced increase of CXCL12 and its release from microglia *via* the TLR4/IB/NF-κB pathway (Ahmed et al., 2012).α-Syn is also a CD36 agonist (Panicker et al., 2019). It has been shown that CD36 and Fyn kinase facilitate the uptake of α-syn by microglia and initiate the assembly of inflammasomes through a protein kinase Cδ-dependent nuclear translocation of NF-κB-p65. Furthermore, uptake of α-syn is reduced in Fyn-deficient microglia and bone marrow-derived macrophages (BMDM) that lack CD36. Therefore, Fyn plays an important role in promoting neuroinflammation in PD (Panicker et al., 2019). In addition, based on a genome-wide association study (GWAS), the Fyn locus is linked to the increased risk of PD (Nalls et al., 2019). Sarkar et al. showed that in PD models, Kv1.3 is elevated. The downstream mediator of the NF-κB and p38 MAPK pathways, the Fyn/PKC signaling cascade, proximally controlled the Kv1.3 upregulation. They showed that Kv1.3 overexpression contributes significantly to neuroinflammation-mediated neurodegeneration in PD models. These findings also point to a possible Kv1.3-mediated signaling pathway which can modulate microglial inflammation in PD (Sarkar et al., 2020b). Sarkar et al. in another study discovered new molecular pathways for α-syn aggregation-induced neuroinflammation. α-Syn upregulated the expression of RNA binding proteins in mouse microglia, implying higher RNA processing and splicing as well as mitochondrial oxidative stress. They also found evidence for decreased microglial progranulin as a new disease mechanism in PD, suggesting that lysosomal dysfunction and autophagy are involved in the disease pathogenesis (Sarkar et al., 2020a). Zhang et al. proposed another novel mechanism. They discovered that mGluR5 was critical in preventing α-syn-induced neuroinflammation. This effect was dependent on the interaction between mGluR5 and α-syn, as well as mGluR5 degradation via the lysosomal pathway induced by α-syn. According to this study, the separation of the mGluR5—α-syn complex in microglia is induced by increased mGluR5 expression (Zhang et al., 2021).

A large number of αsyn transgenic mice models have been developed to replicate a spectrum of clinical and behavioral characteristics of PD and other synucleinopathies. The form of α-syn expressed (wild type vs. mutant) and its promoter-specific expression pattern are the key differences between the existing mouse lines (Kahle, 2008; Chesselet and Richter, 2011; Magen and Chesselet, 2011). A number of mouse lines with α-syn deficiency have also been employed to help researchers understand the roles of this protein in the brain cellular processes (Abeliovich et al., 2000; Specht and Schoepfer, 2004; Kokhan et al., 2012). Transgenic α-syn KO mice (Abeliovich et al., 2000) were initially reported to be unimpaired in spatial memory learning, as demonstrated by the Morris Water Maze (MWM) challenge (Chen et al., 2002). A further study indicated that there are actually cognitive abnormalities in this transgenic mouse model but at more advanced ages compared to the initial report (Kokhan et al., 2012). A likely confounding element in understanding the potential role of α-syn in cognitive dysfunction seems to be the compensatory function of gamma synuclein (γ-syn) in synaptic regulation during the absence of α-syn, resulting in the alleviation of cognitive abnormalities in α-syn-KO mice (Senior et al., 2008). Therefore, γ-syn could perform a compensatory function by restoring cognitive functions in α-syn-KO mice (Hatami and Chesselet, 2015).

A53T α-syn causes neurodegeneration (Giasson et al., 2002; Gu et al., 2010). Mice with A53T α-syn have significant motor impairments, which can lead to paralysis and death (Giasson et al., 2002). In a study by Gu et al. (2010), inflammation and microglial activation were promoted by the A53T α-syn, which caused astrogliosis, particularly in the midbrain, brainstem, and spinal cord. This study also discovered a significant DA neurons loss in the midbrain and motor neurons of the spinal cord in symptomatic mice, which could explain the paralysis characteristics of mutant mice. Furthermore, COX-1-mediated inflammatory pathways can play a role in neurodegeneration, as indicated by the COX-1 inhibitor's ability to lengthen the lifespan of A53T mice. The A53T α-syn mice also developed age-dependent α-syn inclusions, which mimic the pathology seen in people with PD. Overexpression of A53T α-syn inhibited complex I function in DA neurons of transgenic mice (Chinta et al., 2010), depolarized mitochondrial membrane potential, increased ROS in human neuroblastoma cells (Parihar et al., 2009), and induced mitochondrial autophagy in neurons bearing the A53T mutation (Chinta et al., 2010; Choubey et al., 2011). Furthermore, α-syn has been demonstrated to alter mitochondrial motility (Xie and Chung, 2012). One theory for the mechanism underlying the effect of A53T α-syn on mitochondria is that it raises Ca2+ signal in neurons, which has been demonstrated to limit mitochondrial mobility (Yi et al., 2004; Wang and Schwarz, 2009). Previous investigations have suggested that A53T α-syn can create Ca2+ permeable holes in the plasma membrane (Furukawa et al., 2006) and can control Ca2+ entry pathway (Hettiarachchi et al., 2009). Li et al. discovered that A53T α-syn decreased both overall mitochondrial mobility and the fraction of mobile mitochondria. In other words, in A53T α-syn neurons, the percentage of stationary mitochondria rose (Li et al., 2013); therefore, it is probable that A53T α-syn controls syntaphilin or myosin (Kang et al., 2008; Pathak et al., 2010) to improve anchoring of stationary mitochondria. Mice expressing A30P α-syn have failed to exhibit alterations in locomotor activity, and dopamine levels in spite of the buildup of α-syn in various brain regions (Kahle et al., 2001; Yavich et al., 2004; Freichel et al., 2007). Kilpeläinen et al. characterized homozygous double mutant A30P^*^A53T α-syn transgenic mice and reported that these animals did show early onset and age-dependent alterations in striatal dopaminergic function and locomotor activity, as well as formation of α-syn oligomers, suggesting that it could be a useful tool for modeling early onset PD associated with familial SNCA mutations (Kilpeläinen et al., 2019). Thy-1 promoter has been used for producing transgenic α-syn overexpressing mice. The α-syn transgene is widely expressed in these mice, and cytoplasmic and nuclear inclusions containing human α-syn appear in various brain areas, including the cortex, hippocampus, olfactory bulb, and to a lesser extent, the substantia nigra (Cenci and Björklund, 2020).

Our review also highlighted the roles of adaptive immune response in PD models. T lymphocyte infiltration and enhanced MHC II immunoreactivity were seen in MPTP-treated mice, but no B lymphocyte infiltration was reported (Kurkowska-Jastrzebska et al., 1999; Karikari et al., 2022). Furthermore, the injection of regulatory T cells reduced the neurotoxicity of MPTP (Reynolds et al., 2007). In contrast to T lymphocytes' obvious participation in human PD, there is no indication of B lymphocytes' presence in the brains of animal models of PD. It is worth mentioning that mice lacking both B and T lymphocytes were resistant to MPTP toxicity in a recent study employing the MPTP mouse model of PD (Benner et al., 2008). In the past, research on neuroinflammation in AD and PD has primarily focused on aberrant innate immune system activation (Benner et al., 2008; Rodrigues et al., 2014; Caplan and Maguire-Zeiss, 2018; Labzin et al., 2018). Recent data suggests that changes in the adaptive immune response may also play a role in inflammation and neurodegeneration in Alzheimer's disease and age-related synucleinopathies (Kannarkat et al., 2013; Allen Reish and Standaert, 2015; Baird et al., 2019). α-Syn oligomers and fibrils increased the ratio of CD8+ to CD4+ T cells in the CNS and decreased the expression of STAT3, CD25, and CD127 in CD3+CD4+ T cells. CD4+ T cell infiltration into the CNS has also been linked to changes in the phenotype of brain microglia (Olesen et al., 2018). Thus, CD3+CD4+ T cells' homing and tolerance capabilities are affected by α-syn aggregates (Olesen et al., 2018). In acute neurotoxic models of PD, such as MPTP-injected mice, substantial T cell infiltration was detected in the substantia nigra at the first day following MPTP challenge, and gradually decreased and normalized by day 30 (Chandra et al., 2017). In addition to the potentially neurotoxic impacts of cytokines secreted by Th1 or Th17 cells (Park et al., 2017; Storelli et al., 2019), Th2 and Treg cells are considered to suppress innate immune activation in the CNS, indicating that an imbalance in T cell types may cause overactivation of glia and chronic inflammation (Gendelman and Appel, 2011; Olson and Gendelman, 2016; von Euler Chelpin and Vorup-Jensen, 2017). Previous research has revealed that α-syn aggregates are released into the extracellular space under pathological situations (Desplats et al., 2009; Lee et al., 2014; Emmanouilidou and Vekrellis, 2016; Steiner et al., 2018), in which they can potentially stimulate T cells. In the context of MHC class II, two types of antigen-presenting cells are reported to display epitopes originating from the α-syn Y39 region. IL-5 from CD4+ T cells and IFN from CD8+ T cells are the main triggers of this response (Sulzer et al., 2017). As a result, α-syn peptides can behave as antigenic epitopes, activating T cell responses, which could explain the link between PD and specific MHC alleles (Sulzer et al., 2017). Recent research has found that extracellular α-syn has a variety of impacts on CD4+ and CD8+ T cell populations in the peripheral and central nervous systems, implying that α-syn variations affect CD4+ T cell homing and tolerance capacity (Olesen et al., 2018). Another study used a combination of human α-syn PFF and AAV-human-α-syn injections into the rat substantia nigra and found both microglia activation and CD4+ and CD8+ T cell infiltration (Thakur et al., 2017). Furthermore, extracellular α-syn aggregates have been reported to inhibit CD25 expression, which could explain why recently activated T cells in PD have a lower survival potential (Olesen et al., 2018).

It is reported that Fas-deficient animals have less MPTP-induced DA neuron loss (Hayley et al., 2004). While the Fas/FasL pathway has been linked to the removal of activated macrophages and therefore to the resolution of inflammation in the setting of antigen presentation (Ashany et al., 1995), new evidence reveals that this pathway may alternatively generate proinflammatory cytokines in tissue macrophages (Park et al., 2003). As a result, CD4+ Th FasL-mediated activation of microglial cells may play a role in the inflammatory response and degeneration of DA neurons. FasL produced by T cells may potentially play a role in inflammatory responses in astrocytes, which are known to be highly resistant to Fas-mediated cell death and to produce proinflammatory cytokines and chemokines in response to Fas ligation (Choi and Benveniste, 2004). Fas expression has been found to be elevated on these glial cells in the MPTP model (Ferrer et al., 2000; Hayley et al., 2004). Alternatively, cell-to-cell interaction between infiltrating CD4^+^ T cells and DA neurons may cause neuronal death (Giuliani et al., 2003).

Our review unveils the critical role of mast cells in the pathogenesis of PD. As stated above, MPP^+^ treatment induces mast cell activation and a subsequent increase in levels of CCL2, IL-33, and ROS generation (Kempuraj et al., 2016, 2019). This is in line with a review by Sandhu and Kulka (2021) that reported MPP ^+^ is an active metabolite of MPTP that causes activation of mouse bone marrow-derived mast cells (BMMC) and increased release of CCL-2 and MMP-3. Thus, IL-33 secretion by mast cells is increased after MPP^+^ treatment and plays its role through a heterodimeric receptor complex consisting of suppression of tumorigenicity 2 (ST2) and the accessory IL-1 receptor protein (IL-1RAP). The IL-33/ST2 pathway is involved in CNS homeostasis and its pathologies, including neurodegenerative diseases (Sun et al., 2021). Mast cells are a population of IL-33 targeting cells, recognizing it by IL-33 receptor, ST2 (Lunderius-Andersson et al., 2012). Mast cells activation is observed in PD brains and may play role in neuroinflammation in this disease (Kempuraj et al., 2015, 2019).

TG2 is expressed by mast cells in MPTP-treated mice, which can stimulate inflammatory cytokines and neuroinflammation (Hong et al., 2018). A review by Kim et al. (2013) confirms the roles of TG2 in PD and other neurodegenerative diseases. This gene has been reported to encode an enzyme with four different activities, including protein disulfide isomerase, transamidase, protein kinase, and GTPase. Its transamination function can cause a toxic and insoluble aggregation of amyloid and other proteins. In the Lewy bodies, large numbers of isopeptide bonds produced by TG2 were found. The SH-SY5Y neuroblastoma cell line was treated with MPP+, which greatly elevated TG2 activity (Beck et al., 2006; Verhaar et al., 2011). It is established that α-syn is one of TG2's substrates (Junn et al., 2003). TG2 catalyzes the cross-linking of α-synuclein, resulting in the formation of insoluble, high-molecular-weight aggregates. TG2 was recently discovered to be a substrate of PINK1, a PD-associated Ser/Thr protein kinase. PINK1 phosphorylates TG2 directly, increasing protein stability by preventing proteasomal breakdown (Min et al., 2015). As a result, PINK1 regulates TG2 activity, which may be linked to the production of aggresomes in neural cells (Min et al., 2015). According to recent studies, endoplasmic reticulum (ER) dysfunction is a key component of PD development. As a result, proper TG2 function is intimately linked to ER function. In MPP-treated SH-SY5Y cells, for example, biochemical contact and colocalization between TG2 and ER were detected (Verhaar et al., 2012). In a separate investigation, it was discovered that the localization of TG2 to the granular ER compartment in the PD brain is highly selective for stressed and melanized neurons (Wilhelmus et al., 2011). In this regard, TG2 inhibitors may be a promising therapy for alleviating the brain diseases which TG2 plays role in (Min and Chung, 2018). Additionally, inhibition of pKr-2 is identified as a potential therapeutic strategy in PD since pKr-2 treatment of rats causes an increase in microglial TLR4, which is essential for their immune activation (Shin et al., 2015). Furthermore, TLR4 agonists can cause necroptosis, which leads to cell death and release of their intracellular contents triggering innate immune responses and neuroinflammation (Yu et al., 2021); therefore, TLR4 antagonists could have therapeutic potential in PD and other neuroinflammatory disorders as already summarized by Leitner et al. (2019). Suppression of PGE2 is another potential treatment strategy in PD, which is supported by observations that the DA toxin 6-OHDA upregulates mPGES-1 and triggers PGE2-dependent death of DA neurons (Ikeda-Matsuo et al., 2019). This therapeutic approach has been reviewed by Singh et al. (2021).

Aging is a physiological challenge that all organisms face throughout time, and it is also the leading risk factor for neurodegenerative illnesses. Therefore. effects of aging on the neuroinflammation within PD mice models should be considered. Zhao et al. showed that in aged mice, compared to young mice, behavioral performance decreased and DA neurons were depleted, which was followed by increased expression of pro-inflammatory factors (TLR2, p-NF-κB-p65, IL-1β, and TNF-α), as well as the pro-oxidative stress factor gp91phox. The inflammatory M1 microglia were increased by aging, and the equilibrium between oxidation and anti-oxidantswas disrupted. In LPS-treated, aged mice, poor behavioral performance and loss of DA neurons were observed, as well as upregulated TLR2, p-NF-κB-p65, IL-1β, TNF-α, iNOS, and gp91phox (Zhao et al., 2018). Also, Yao and Zhao demonstrated that in a MPTP-PD mouse model, aging enhanced M1 microglia activation while inhibiting M2 microglia activation in the substantia nigra, which was associated with an increase in proinflammatory cytokines TNF-α and IL-1β (Yao and Zhao, 2018).

We also reviewed cell models of PD used in the included studies. Rat/mice primary neuron/glia, BV2 murine microglial cells, and SH-SY5Y human neuroblastoma cell line were among the most common cell lines used for creating *in vitro* PD models. PD cell models have some advantages in comparison with animal models. First, PD-related genes can be efficiently overexpressed or knocked out in cultured cells. Second, in dopamine-producing cell lines, both MPP+ and 6-OHDA can be utilized to trigger cell death. Other benefits of these models include their unlimited proliferation which allows high-throughput experimentation with a wide range of experimental techniques and endpoints; homogeneity of cell populations which leads to high reproducibility; and the fact that some cell lines such as SH-SY5Y express important enzymes for dopamine metabolism and synapse formation (Han et al., 2003; Schildknecht et al., 2009; Lopes et al., 2010; Scholz et al., 2011; Thomas et al., 2013). Their main disadvantage is high proliferative capacity, which differs from neurons that do not divide. In comparison to primary neurons and organotypic cultures, immortalized cells are not only unable to replicate the appearance and physiology of a neuronal cell, but they also do not express many of the synaptic proteins. In addition, continuous proliferation induces a selection pressure that favors mutations that improve proliferation and survival, causing succeeding generations of cell lines to lose their DA phenotype in comparison to their parental lines. As a result, after repeated passaging, many cell lines become inappropriate for usage (Lopes et al., 2017a). Despite having important DA traits, SH-SY5Y cells lack neuronal characteristics. This cell line is in the early phases of neuronal development, with low numbers of neuronal markers. Furthermore, their oncogenic characteristics and persistent multiplication are incompatible with neurons (Gilany et al., 2008; Filograna et al., 2015; Lopes et al., 2017a). Both genetic and toxin-based techniques have been used to recreate PD pathology in this cell model, with 6-OHDA being the most widely used toxin. The majority of these investigations are concerned with achieving neuroprotection in cells exposed to 6-OHDA (Wei et al., 2015; Lin and Tsai, 2017). Undifferentiated SH-SY5Y cells have also been used to investigate the mechanisms of 6-OHDA toxicity (Soto-Otero et al., 2000; Izumi et al., 2005; Xicoy et al., 2017). 6-OHDA is taken up by DA neurons *via* DAT transporter and induces considerable oxidative stress. Undifferentiated cells do not mimic the 6-OHDA-induced cell death mechanisms that occur *in vivo* because they only express modest amounts of DAT (Lopes et al., 2017b).

## Conclusion

In the current systematic review, we have collected data identifying the importance of several neuroinflammatory pathways and molecular mechanisms in the pathogenesis of PD. We conclude that neuroinflammation plays a role in both the initiation and progression of PD. We illustrate that neuroinflammatory reactions in PD models can be induced by various factors including MPTP, α-syn, 6-OHDA, and pKr-2, while other mechanisms, such as TLR2, NLRP3, IL-1β, TNF-α, NF-κB pathway, HMBG1, ROS production, CD36, Fyn, mast cells, ASK1, Lcn-2, TG2, CCL2, IL-33, TLR4, mPGES-1, and PGE2, contribute to the establishment and progression of the pathogenetic mechanisms in these models.

In addition, we identify several potential therapeutic approaches that may be effective in PD by alleviating neuroinflammation. They include miR-124 and miR-7 as well as inhibitors of NLRP3 inflammasomes, HMBG1, and TG2. We also acknowledge limitations to our work, which include very limited research on some of the mechanisms we review and a shortage of other comprehensive systematic reviews and meta-analyses that could be used to further validate our conclusions. Further research is needed to understand other neuroinflammatory mechanisms involved in the pathogenesis of PD and to develop new therapeutic approaches targeting them. By evaluating the relative impact of each factor and determining their collective contribution to neuroinflammation in PD, multitargeted therapeutic approaches could be developed that will hopefully solve the puzzle of PD, which currently lacks effective treatments.

## Data Availability Statement

Publicly available datasets were analyzed in this study. This data can be found at: https://pubmed.ncbi.nlm.nih.gov/.

## Author Contributions

MF, KV, and SY contributed to the conception and design of the study. MRT and FS contributed to the supervision of the manuscript. MD organized the database. AK edited the paper scientifically. All authors wrote the first draft of the manuscript, wrote sections of the manuscript, contributed to manuscript revision, read, and approved the submitted version.

## Funding

This study was related to the Project (No. 1399/61320) from Student Research Committee, Shahid Beheshti University of Medical Sciences, Tehran, Iran.

## Conflict of Interest

The authors declare that the research was conducted in the absence of any commercial or financial relationships that could be construed as a potential conflict of interest.

## Publisher's Note

All claims expressed in this article are solely those of the authors and do not necessarily represent those of their affiliated organizations, or those of the publisher, the editors and the reviewers. Any product that may be evaluated in this article, or claim that may be made by its manufacturer, is not guaranteed or endorsed by the publisher.

## References

[B1] Abeliovich A. Schmitz Y. Fariñas I. Choi-Lundberg D. Ho W.-H. Castillo P. E. . (2000). Mice lacking α-synuclein display functional deficits in the nigrostriatal dopamine system. Neuron 25, 239–252. 10.1016/S0896-6273(00)80886-7 10707987

[B2] Ahmed I. Tamouza R. Delord M. Krishnamoorthy R. Tzourio C. Mulot C. . (2012). Association between Parkinson's disease and the HLA-DRB1 locus. Mov. Disord. 27, 1104–1110. 10.1002/mds.25035 22807207

[B3] Allen Reish H. E. Standaert D. G. (2015). Role of α-synuclein in inducing innate and adaptive immunity in Parkinson disease. J. Parkinsons Dis. 5, 1–19. 10.3233/JPD-140491 25588354 PMC4405142

[B4] Ambrosi G. Kustrimovic N. Siani F. Rasini E. Cerri S. Ghezzi C. . (2017). Complex changes in the innate and adaptive immunity accompany progressive degeneration of the nigrostriatal pathway induced by intrastriatal injection of 6-hydroxydopamine in the rat. Neurotox. Res. 32, 71–81. 10.1007/s12640-017-9712-2 28285346

[B5] Ashany D. Song X. Lacy E. Nikolic-Zugic J. Friedman S. M. Elkon K. B. (1995). Th1 CD4+ lymphocytes delete activated macrophages through the Fas/APO-1 antigen pathway. Proc. Natl. Acad. Sci. U.S.A. 92, 11225–11229. 10.1073/pnas.92.24.11225 7479970 PMC40604

[B6] Bae E. Lee H. Jang Y. Michael S. Masliah E. Min D. . (2014). Phospholipase D1 regulates autophagic flux and clearance of α-synuclein aggregates. Cell Death Differ. 21, 1132–1141. 10.1038/cdd.2014.30 24632948 PMC4207479

[B7] Baeuerle P. A. Baltimore D. (1996). NF-kappa B: ten years after. Cell 87, 13–20. 10.1016/S0092-8674(00)81318-5 8858144

[B8] Baird J. K. Bourdette D. Meshul C. K. Quinn J. F. (2019). The key role of T cells in Parkinson's disease pathogenesis and therapy. Parkinsonism Relat. Disord. 60, 25–31. 10.1016/j.parkreldis.2018.10.029 30404763

[B9] Baltic S. Perovic M. Mladenovic A. Raicevic N. Ruzdijic S. Rakic L. . (2004). α-Synuclein is expressed in different tissues during human fetal development. J. Mol. Neurosci. 22, 199–203. 10.1385/JMN:22:3:199 14997013

[B10] Bartel D. P.. (2004). MicroRNAs: genomics, biogenesis, mechanism, and function. Cell 116, 281–297. 10.1016/S0092-8674(04)00045-5 14744438

[B11] Bauernfeind F. G. Horvath G. Stutz A. Alnemri E. S. MacDonald K. Speert D. . (2009). Cutting edge: NF-κB activating pattern recognition and cytokine receptors license NLRP3 inflammasome activation by regulating NLRP3 expression. J. Immunol. 183, 787–791. 10.4049/jimmunol.0901363 19570822 PMC2824855

[B12] Beck K. E. De Girolamo L. A. Griffin M. Billett E. E. (2006). The role of tissue transglutaminase in 1-methyl-4-phenylpyridinium (MPP+)-induced toxicity in differentiated human SH-SY5Y neuroblastoma cells. Neurosci. Lett. 405, 46–51. 10.1016/j.neulet.2006.06.061 16876317

[B13] Bendor J. T. Logan T. P. Edwards R. H. (2013). The function of α-synuclein. Neuron 79, 1044–1066. 10.1016/j.neuron.2013.09.004 24050397 PMC3866954

[B14] Benner E. J. Banerjee R. Reynolds A. D. Sherman S. Pisarev V. M. Tsiperson V. . (2008). Nitrated alpha-synuclein immunity accelerates degeneration of nigral dopaminergic neurons. PLoS ONE 3, e1376. 10.1371/journal.pone.0001376 18167537 PMC2147051

[B15] Berry C. La Vecchia C. Nicotera P. (2010). Paraquat and Parkinson's disease. Cell Death Differ. 17, 1115–1125. 10.1038/cdd.2009.217 20094060

[B16] Betarbet R. Sherer T. B. Greenamyre J. T. (2002). Animal models of Parkinson's disease. Bioessays 24, 308–318. 10.1002/bies.10067 11948617

[B17] Bieganowska K. Członkowska A. Bidziński A. Mierzewska H. Korlak J. (1993). Immunological changes in the MPTP-induced Parkinson's disease mouse model. J. Neuroimmunol. 42, 33–37. 10.1016/0165-5728(93)90209-H 8423206

[B18] Block M. L. Zecca L. Hong J.-S. (2007). Microglia-mediated neurotoxicity: uncovering the molecular mechanisms. Nat. Rev. Neurosci. 8, 57–69. 10.1038/nrn2038 17180163

[B19] Boireau A. Bordier F. Dubédat P. Doble A. (1995). Methamphetamine and dopamine neurotoxicity: differential effects of agents interfering with glutamatergic transmission. Neurosci. Lett. 195, 9–12. 10.1016/0304-3940(95)11765-O 7478259

[B20] Brochard V. Combadière B. Prigent A. Laouar Y. Perrin A. Beray-Berthat V. . (2009). Infiltration of CD4+ lymphocytes into the brain contributes to neurodegeneration in a mouse model of Parkinson disease. J. Clin. Invest. 119, 182–192. 10.1172/JCI36470 19104149 PMC2613467

[B21] Burré J. Sharma M. Südhof T. C. (2014). α-Synuclein assembles into higher-order multimers upon membrane binding to promote SNARE complex formation. Proc. Natl. Acad. Sci. U.S.A. 111, E4274–E4283. 10.1073/pnas.1416598111 25246573 PMC4210039

[B22] Burré J. Sharma M. Südhof T. C. (2015). Definition of a molecular pathway mediating α-synuclein neurotoxicity. J. Neurosci. 35, 5221–5232. 10.1523/JNEUROSCI.4650-14.2015 25834048 PMC4380997

[B23] Burré J. Sharma M. Südhof T. C. (2018). Cell Biology and Pathophysiology of α-Synuclein. Cold Spring Harb. Perspect. Med. 8, ea024091. 10.1101/cshperspect.a024091 PMC5519445 28108534

[B24] Burré J. Sharma M. Tsetsenis T. Buchman V. Etherton M. R. Südhof T. C. (2010). Alpha-synuclein promotes SNARE-complex assembly *in vivo* and *in vitro*. Science 329, 1663–1667. 10.1126/science.1195227 20798282 PMC3235365

[B25] Campolo M. Paterniti I. Siracusa R. Filippone A. Esposito E. Cuzzocrea S. (2019). TLR4 absence reduces neuroinflammation and inflammasome activation in Parkinson's diseases *in vivo* model. Brain Behav. Immun. 76, 236–247. 10.1016/j.bbi.2018.12.003 30550933

[B26] Cao S. Standaert D. G. Harms A. S. (2012). The gamma chain subunit of Fc receptors is required for alpha-synuclein-induced pro-inflammatory signaling in microglia. J. Neuroinflammation 9, 259. 10.1186/1742-2094-9-259 23186369 PMC3526448

[B27] Caplan I. F. Maguire-Zeiss K. A. (2018). Toll-like receptor 2 signaling and current approaches for therapeutic modulation in synucleinopathies. Front. Pharmacol. 9, 417. 10.3389/fphar.2018.00417 PMC5945810 29780321

[B28] Cenci M. A. Björklund A. (2020). Animal models for preclinical Parkinson's research: an update and critical appraisal. Prog. Brain Res. 252, 27–59. 10.1016/bs.pbr.2020.02.003 32247366

[B29] Chandra G. Roy A. Rangasamy S. B. Pahan K. (2017). Induction of adaptive immunity leads to nigrostriatal disease progression in MPTP mouse model of Parkinson's disease. J. Immunol. 198, 4312–4326. 10.4049/jimmunol.1700149 28446566 PMC5467696

[B30] Chen M. J. Yap Y. W. Choy M. S. Koh C. H. V. Seet S. J. Duan W. . (2006). Early induction of calpains in rotenone-mediated neuronal apoptosis. Neurosci. Lett. 397, 69–73. 10.1016/j.neulet.2005.12.011 16412576

[B31] Chen P. E. Specht C. G. Morris R. G. Schoepfer R. (2002). Spatial learning is unimpaired in mice containing a deletion of the alpha-synuclein locus. Euro. J. Neurosci. 16, 154–158. 10.1046/j.1460-9568.2002.02062.x 12153541

[B32] Chesselet M.-F. Richter F. (2011). Modelling of Parkinson's disease in mice. Lancet Neurol. 10, 1108–1118. 10.1016/S1474-4422(11)70227-7 22094131

[B33] Chesselet M.-F. Richter F. Zhu C. Magen I. Watson M. B. Subramaniam S. R. (2012). A progressive mouse model of Parkinson's disease: the Thy1-aSyn (“Line 61”) mice. Neurotherapeutics 9, 297–314. 10.1007/s13311-012-0104-2 22350713 PMC3337020

[B34] Chinta S. J. Mallajosyula J. K. Rane A. Andersen J. K. (2010). Mitochondrial α-synuclein accumulation impairs complex I function in dopaminergic neurons and results in increased mitophagy *in vivo*. Neurosci Lett. 486, 235–239. 10.1016/j.neulet.2010.09.061 20887775 PMC2967673

[B35] Choi C. Benveniste E. N. (2004). Fas ligand/Fas system in the brain: regulator of immune and apoptotic responses. Brain Res. Rev. 44, 65–81. 10.1016/j.brainresrev.2003.08.007 14739003

[B36] Choi I. Zhang Y. Seegobin S. P. Pruvost M. Wang Q. Purtell K. . (2016). Microglia clear neuron-released α-synuclein via selective autophagy and prevent neurodegeneration. Nat. Commun. 11, 1386. 10.1038/s41467-020-15119-w PMC7069981 32170061

[B37] Choi W.-S. Kim H.-W. Xia Z. (2015). JNK inhibition of VMAT2 contributes to rotenone-induced oxidative stress and dopamine neuron death. Toxicology 328, 75–81. 10.1016/j.tox.2014.12.005 25496994 PMC4308505

[B38] Choubey V. Safiulina D. Vaarmann A. Cagalinec M. Wareski P. Kuum M. . (2011). Mutant A53T alpha-synuclein induces neuronal death by increasing mitochondrial autophagy. J. Biol. Chem. 286, 10814–10824. 10.1074/jbc.M110.132514 21252228 PMC3060532

[B39] Cleeter M. Cooper J. Schapira A. (1992). Irreversible inhibition of mitochondrial complex I by 1-methyl-4-phenylpyridinium: evidence for free radical involvement. J. Neurochem. 58, 786–789. 10.1111/j.1471-4159.1992.tb09789.x 1729421

[B40] Conde M. A. Alza N. P. González P. A. I. Bilbao P. G. S. Campos S. S. Uranga R. M. . (2018). Phospholipase D1 downregulation by α-synuclein: Implications for neurodegeneration in Parkinson's disease. Biochim. Biophys. Acta 1863, 639–650. 10.1016/j.bbalip.2018.03.006 29571767

[B41] Cunningham C. Dunne A. Lopez-Rodriguez A. B. (2019). Astrocytes: heterogeneous and dynamic phenotypes in neurodegeneration and innate immunity. Neuroscientist 25, 455–474. 10.1177/1073858418809941 30451065 PMC6525076

[B42] Członkowska A. Kohutnicka M. Kurkowska-Jastrzebska I. Członkowski A. (1996). Microglial reaction in MPTP (1-methyl-4-phenyl-1,2,3,6-tetrahydropyridine) induced Parkinson's disease mice model. Neurodegeneration. 5, 137–143. 10.1006/neur.1996.0020 8819134

[B43] Dauer W. Kholodilov N. Vila M. Trillat A.-C. Goodchild R. Larsen K. E. . (2002). Resistance of alpha -synuclein null mice to the parkinsonian neurotoxin MPTP. Proc. Natl. Acad. Sci. U.S.A. 99, 14524–14529. 10.1073/pnas.172514599 12376616 PMC137916

[B44] Dekens D. W. Eisel U. L. M. Gouweleeuw L. Schoemaker R. G. De Deyn P. P. Naud,é P. J. W. (2021). Lipocalin 2 as a link between ageing, risk factor conditions and age-related brain diseases. Ageing Res. Rev. 70, 101414. 10.1016/j.arr.2021.101414 34325073

[B45] Desplats P. Lee H. J. Bae E. J. Patrick C. Rockenstein E. Crews L. . (2009). Inclusion formation and neuronal cell death through neuron-to-neuron transmission of alpha-synuclein. Proc. Natl. Acad. Sci. U.S.A. 106, 13010–13015. 10.1073/pnas.0903691106 19651612 PMC2722313

[B46] Deumens R. Blokland A. Prickaerts J. (2002). Modeling parkinson's disease in rats: an evaluation of 6-OHDA lesions of the nigrostriatal pathway. Exp. Neurol. 175, 303–317. 10.1006/exnr.2002.7891 12061862

[B47] Earls R. H. Menees K. B. Chung J. Barber J. Gutekunst C. A. Hazim M. G. . (2019). Intrastriatal injection of preformed alpha-synuclein fibrils alters central and peripheral immune cell profiles in non-transgenic mice. J. Neuroinflammation 16, 250. 10.1186/s12974-019-1636-8 31796095 PMC6889316

[B48] Emmanouilidou E. Vekrellis K. (2016). Exocytosis and spreading of normal and aberrant α-synuclein. Brain Pathol. 26, 398–403. 10.1111/bpa.12373 26940375 PMC8029167

[B49] Engblom D. Saha S. Engström L. Westman M. Audoly L. P. Jakobsson P. J. . (2003). Microsomal prostaglandin E synthase-1 is the central switch during immune-induced pyresis. Nat. Neurosci. 6, 1137–1138. 10.1038/nn1137 14566340

[B50] Falkenburger B. Schulz J. (2006). Limitations of cellular models in Parkinson's disease research. Parkinsons Dis. Relat. Disord. 261–268. 10.1007/978-3-211-45295-0_40 17017539

[B51] Faull R. Laverty R. (1969). Changes in dopamine levels in the corpus striatum following lesions in the substantia nigra. Exp. Neurol. 23, 332–340. 10.1016/0014-4886(69)90081-8 5767257

[B52] Fernagut P.-O. Chesselet M.-F. (2004). Alpha-synuclein and transgenic mouse models. Neurobiol. Dis. 17, 123–130. 10.1016/j.nbd.2004.07.001 15474350

[B53] Fernagut P. O. Hutson C. B. Fleming S. M. Tetreaut N. A. Salcedo J. Masliah E. . (2007). Behavioral and histopathological consequences of paraquat intoxication in mice: effects of α-synuclein over-expression. Synapse 61, 991–1001. 10.1002/syn.20456 17879265 PMC3097512

[B54] Ferrari C. C. Godoy M. C. P. Tarelli R. Chertoff M. Depino A. M. Pitossi F. J. (2006). Progressive neurodegeneration and motor disabilities induced by chronic expression of IL-1β in the substantia nigra. Neurobiol. Dis. 24, 183–193. 10.1016/j.nbd.2006.06.013 16901708

[B55] Ferrer I. Blanco R. Cutillas B. Ambrosio S. (2000). Fas and Fas-L expression in Huntington's disease and Parkinson's disease. Neuropathol. Appl. Neurobiol. 26, 424–433. 10.1046/j.1365-2990.2000.00267.x 11054182

[B56] Filograna R. Civiero L. Ferrari V. Codolo G. Greggio E. Bubacco L. . (2015). Analysis of the catecholaminergic phenotype in human SH-SY5Y and BE (2)-M17 neuroblastoma cell lines upon differentiation. PLoS ONE 10, e0136769. 10.1371/journal.pone.0136769 26317353 PMC4552590

[B57] Fleming S. M. Tetreault N. A. Mulligan C. K. Hutson C. B. Masliah E. Chesselet M. F. (2008). Olfactory deficits in mice overexpressing human wildtype alpha-synuclein. Eur. J. Neurosci. 28, 247–256. 10.1111/j.1460-9568.2008.06346.x 18702696 PMC3108548

[B58] Freichel C. Neumann M. Ballard T. Müller V. Woolley M. Ozmen L. . (2007). Age-dependent cognitive decline and amygdala pathology in α-synuclein transgenic mice. Neurobiol. Aging 28, 1421–1435. 10.1016/j.neurobiolaging.2006.06.013 16872721

[B59] Furukawa K. Matsuzaki-Kobayashi M. Hasegawa T. Kikuchi A. Sugeno N. Itoyama Y. . (2006). Plasma membrane ion permeability induced by mutant alpha-synuclein contributes to the degeneration of neural cells. J. Neurochem. 97, 1071–1077. 10.1111/j.1471-4159.2006.03803.x 16606366

[B60] Gardai S. J. Mao W. Schüle B. Babcock M. Schoebel S. Lorenzana C. . (2013). Elevated alpha-synuclein impairs innate immune cell function and provides a potential peripheral biomarker for Parkinson's disease. PLoS ONE. 8, e71634. 10.1371/journal.pone.0071634 PMC3751933 24058406

[B61] Gendelman H. E. Appel S. H. (2011). Neuroprotective activities of regulatory T cells. Trends Mol. Med. 17, 687–688. 10.1016/j.molmed.2011.08.005 21996344 PMC5892451

[B62] Giasson B. I. Duda J. E. Quinn S. M. Zhang B. Trojanowski J. Q. Lee V. M. (2002). Neuronal alpha-synucleinopathy with severe movement disorder in mice expressing A53T human alpha-synuclein. Neuron 34, 521–533. 10.1016/S0896-6273(02)00682-7 12062037

[B63] Gilany K. Van Elzen R. Mous K. Coen E. Van Dongen W. Vandamme S. . (2008). The proteome of the human neuroblastoma cell line SH-SY5Y: an enlarged proteome. Biochim. Biophys. Acta 1784, 983–985. 10.1016/j.bbapap.2008.03.003 18402783

[B64] Gispert S. Turco D. D. Garrett L. Chen A. Bernard D. J. Hamm-Clement J. . (2003). Transgenic mice expressing mutant A53T human alpha-synuclein show neuronal dysfunction in the absence of aggregate formation. Mol. Cell. Neurosci. 24, 419–429. 10.1016/S1044-7431(03)00198-2 14572463

[B65] Giuliani F. Goodyer C. G. Antel J. P. Yong V. W. (2003). Vulnerability of human neurons to T cell-mediated cytotoxicity. J. Immunol. 171, 368–379. 10.4049/jimmunol.171.1.368 12817020

[B66] Goedert M.. (2001). Alpha-synuclein and neurodegenerative diseases. Nat. Rev. Neurosci. 2, 492–501. 10.1038/35081564 11433374

[B67] Greenamyre J. T. Betarbet R. Sherer T. B. (2003). The rotenone model of Parkinson's disease: genes, environment and mitochondria. Parkinsonism Relat. Disord. 9, 59–64. 10.1016/S1353-8020(03)00023-3 12915069

[B68] Gu X. L. Long C. X. Sun L. Xie C. Lin X. Cai H. (2010). Astrocytic expression of Parkinson's disease-related A53T alpha-synuclein causes neurodegeneration in mice. Mol. Brain 3, 12. 10.1186/1756-6606-3-12 20409326 PMC2873589

[B69] Guo X. Namekata K. Kimura A. Harada C. Harada T. (2017). ASK1 in neurodegeneration. Adv. Biol. Regul. 66, 63–71. 10.1016/j.jbior.2017.08.003 28882588

[B70] Gustot A. Gallea J. I. Sarroukh R. Celej M. S. Ruysschaert J.-M. Raussens V. (2015). Amyloid fibrils are the molecular trigger of inflammation in Parkinson's disease. Biochem. J. 471, 323–333. 10.1042/BJ20150617 26272943

[B71] Hamza T. H. Zabetian C. P. Tenesa A. Laederach A. Montimurro J. Yearout D. . (2010). Common genetic variation in the HLA region is associated with late-onset sporadic Parkinson's disease. Nat. Genet. 42, 781–785. 10.1038/ng.642 20711177 PMC2930111

[B72] Han B. S. Hong H.-S. Choi W.-S. Markelonis G. J. Oh T. H. Oh Y. J. (2003). Caspase-dependent and-independent cell death pathways in primary cultures of mesencephalic dopaminergic neurons after neurotoxin treatment. J. Neurosci. 23, 5069–5078. 10.1523/JNEUROSCI.23-12-05069.2003 12832530 PMC6741197

[B73] Harms A. S. Ferreira S. A. Romero-Ramos M. (2021). Periphery and brain, innate and adaptive immunity in Parkinson's disease. Acta Neuropathol. 141, 527–545. 10.1007/s00401-021-02268-5 33555429 PMC7952334

[B74] Hasegawa E. Takeshige K. Oishi T. Murai Y. Minakami S. (1990). 1-Methyl-4-phenylpyridinium (MPP+) induces NADH-dependent superoxide formation and enhances NADH-dependent lipid peroxidation in bovine heart submitochondrial particles. Biochem. Biophys. Res. Commun. 170, 1049–1055. 10.1016/0006-291X(90)90498-C 2167668

[B75] Hatami A. Chesselet M. F. (2015). Transgenic rodent models to study alpha-synuclein pathogenesis, with a focus on cognitive deficits. Curr. Top. Behav. Neurosci. 22, 303–330. 10.1007/7854_2014_355 25218491

[B76] Hawk B. J. Khounlo R. Shin Y.-K. (2019). Alpha-synuclein continues to enhance SNARE-dependent vesicle docking at exorbitant concentrations. Front. Neurosci. 13, 216. 10.3389/fnins.2019.00216 PMC6437117 30949020

[B77] Hayley S. Crocker S. J. Smith P. D. Shree T. Jackson-Lewis V. Przedborski S. . (2004). Regulation of dopaminergic loss by Fas in a 1-methyl-4-phenyl-1,2,3,6-tetrahydropyridine model of Parkinson's disease. J. Neurosci. 24, 2045–2053. 10.1523/JNEUROSCI.4564-03.2004 14985447 PMC6730390

[B78] Henn A. Lund S. Hedtjärn M. Schrattenholz A. Pörzgen P. Leist M. (2009). The suitability of BV2 cells as alternative model system for primary microglia cultures or for animal experiments examining brain inflammation. ALTEX 26, 83–94. 10.14573/altex.2009.2.83 19565166

[B79] Henry C. J. Huang Y. Wynne A. M. Godbout J. P. (2009). Peripheral lipopolysaccharide (LPS) challenge promotes microglial hyperactivity in aged mice that is associated with exaggerated induction of both pro-inflammatory IL-1β and anti-inflammatory IL-10 cytokines. Brain Behav. Immun. 23, 309–317. 10.1016/j.bbi.2008.09.002 18814846 PMC2692986

[B80] Hettiarachchi N. T. Parker A. Dallas M. L. Pennington K. Hung C. C. Pearson H. A. . (2009). alpha-Synuclein modulation of Ca2+ signaling in human neuroblastoma (SH-SY5Y) cells. J. Neurochem. 111, 1192–1201. 10.1111/j.1471-4159.2009.06411.x 19860837

[B81] Hirsch E. C. Hunot S. (2009). Neuroinflammation in Parkinson's disease: a target for neuroprotection? Lancet Neurol. 8, 382–397. 10.1016/S1474-4422(09)70062-6 19296921

[B82] Hirsch E. C. Vyas S. Hunot S. (2012). Neuroinflammation in Parkinson's disease. Parkinsonism Relat. Disord. 18, S210–S212. 10.1016/S1353-8020(11)70065-7 22166438

[B83] Hong G. U. Cho J. W. Kim S. Y. Shin J. H. Ro J. Y. (2018). Inflammatory mediators resulting from transglutaminase 2 expressed in mast cells contribute to the development of Parkinson's disease in a mouse model. Toxicol. Appl. Pharmacol. 358, 10–22. 10.1016/j.taap.2018.09.003 30195017

[B84] Hong-rong X. I. E. Lin-sen H. U. Guo-yi L. I. (2010). SH-SY5Y human neuroblastoma cell line: *in vitro* cell model of dopaminergic neurons in Parkinson's disease. Chin. Med. J. 123, 1086–1092. 10.3760/cma.j.issn.0366-6999.2010.08.021 20497720

[B85] Hooijmans C. R. Rovers M. M. De Vries R. B. Leenaars M. Ritskes-Hoitinga M. Langendam M. W. (2014). SYRCLE's risk of bias tool for animal studies. BMC Med. Res. Methodol. 14, 43. 10.1186/1471-2288-14-43 PMC4230647 24667063

[B86] Horvath I. Iashchishyn I. A. Moskalenko R. A. Wang C. Wärmländer S. Wallin C. . (2018). Co-aggregation of pro-inflammatory S100A9 with α-synuclein in Parkinson's disease: *ex vivo* and *in vitro* studies. J. Neuroinflammation 15, 172. 10.1186/s12974-018-1210-9 29866153 PMC5987543

[B87] Huang J. Yang J. Shen Y. Jiang H. Han C. Zhang G. . (2017). HMGB1 mediates autophagy dysfunction via perturbing beclin1-Vps34 complex in dopaminergic cell model. Front. Mol. Neurosci. 10, 13. 10.3389/fnmol.2017.00013 PMC5281633 28197072

[B88] Huang M. Guo M. Wang K. Wu K. Li Y. Tian T. . (2020). HMGB1 mediates paraquat-induced neuroinflammatory responses via activating RAGE signaling pathway. Neurotox. Res. 37, 913–925. 10.1007/s12640-019-00148-1 31858421

[B89] Huang M. Wang B. Li X. Fu C. Wang C. Kang X. (2019). α-synuclein: a multifunctional player in exocytosis, endocytosis, vesicle recycling. Front. Neurosci. 13, 28. 10.3389/fnins.2019.00028 PMC6360911 30745863

[B90] Hughes C. D. Choi M. L. Ryten M. Hopkins L. Drews A. Botía J. A. . (2019). Picomolar concentrations of oligomeric alpha-synuclein sensitizes TLR4 to play an initiating role in Parkinson's disease pathogenesis. Acta Neuropathol. 137, 103–120. 10.1007/s00401-018-1907-y 30225556 PMC6338693

[B91] Iba M. Kim C. Sallin M. Kwon S. Verma A. Overk C. . (2020). Neuroinflammation is associated with infiltration of T cells in Lewy body disease and α-synuclein transgenic models. J. Neuroinflammation 17, 214–214. 10.1186/s12974-020-01888-0 32680537 PMC7368752

[B92] Ikeda-Matsuo Y. Miyata H. Mizoguchi T. Ohama E. Naito Y. Uematsu S. . (2019). Microsomal prostaglandin E synthase-1 is a critical factor in dopaminergic neurodegeneration in Parkinson's disease. Neurobiol. Dis. 124, 81–92. 10.1016/j.nbd.2018.11.004 30423474

[B93] Ishola I. Chaturvedi J. Rai S. Rajasekar N. Adeyemi O. Shukla R. . (2013). Evaluation of amentoflavone isolated from Cnestis ferruginea Vahl ex DC (*Connaraceae*) on production of inflammatory mediators in LPS stimulated rat astrocytoma cell line (C6) and THP-1 cells. J. Ethnopharmacol. 146, 440–448. 10.1016/j.jep.2012.12.015 23376104

[B94] Ishola I. O. Akinyede A. Adeluwa T. Micah C. (2018). Novel action of vinpocetine in the prevention of paraquat-induced parkinsonism in mice: involvement of oxidative stress and neuroinflammation. Metab. Brain Dis. 33, 1493–1500. 10.1007/s11011-018-0256-9 29855979

[B95] Izumi Y. Sawada H. Sakka N. Yamamoto N. Kume T. Katsuki H. . (2005). p-Quinone mediates 6-hydroxydopamine-induced dopaminergic neuronal death and ferrous iron accelerates the conversion of p-quinone into melanin extracellularly. J. Neurosci. Res. 79, 849–860. 10.1002/jnr.20382 15712215

[B96] Jarrott B. Williams S. J. (2016). Chronic brain inflammation: the neurochemical basis for drugs to reduce inflammation. Neurochem. Res. 41, 523–533. 10.1007/s11064-015-1661-7 26177578

[B97] Javed H. Thangavel R. Selvakumar G. P. Dubova I. Schwartz N. Ahmed M. E. . (2020). NLRP3 inflammasome and glia maturation factor coordinately regulate neuroinflammation and neuronal loss in MPTP mouse model of Parkinson's disease. Int. Immunopharmacol. 83, 106441. 10.1016/j.intimp.2020.106441 PMC7255416 32259702

[B98] Jo M. G. Ikram M. Jo M. H. Yoo L. Chung K. C. Nah S. Y. . (2019). Gintonin mitigates MPTP-induced loss of nigrostriatal dopaminergic neurons and accumulation of α-synuclein via the Nrf2/HO-1 pathway. Mol. Neurobiol. 56, 39–55. 10.1007/s12035-018-1020-1 29675576

[B99] Junn E. Ronchetti R. D. Quezado M. M. Kim S. Y. Mouradian M. M. (2003). Tissue transglutaminase-induced aggregation of alpha-synuclein: Implications for lewy body formation in Parkinson's disease and dementia with lewy bodies. Proc. Natl. Acad. Sci. U.S.A. 100, 2047–2052. 10.1073/pnas.0438021100 12576551 PMC149956

[B100] Kahle P. J.. (2008). α-Synucleinopathy models and human neuropathology: similarities and differences. Acta Neuropathol. 115, 87–95. 10.1007/s00401-007-0302-x 17932682

[B101] Kahle P. J. Neumann M. Ozmen L. Müller V. Odoy S. Okamoto N. . (2001). Selective insolubility of α-synuclein in human lewy body diseases is recapitulated in a transgenic mouse model. Am. J. Pathol. 159, 2215–2225. 10.1016/S0002-9440(10)63072-6 11733371 PMC1850592

[B102] Kamei D. Yamakawa K. Takegoshi Y. Mikami-Nakanishi M. Nakatani Y. Oh-Ishi S. . (2004). Reduced pain hypersensitivity and inflammation in mice lacking microsomal prostaglandin e synthase-1. J. Biol. Chem. 279, 33684–33695. 10.1074/jbc.M400199200 15140897

[B103] Kang J. S. Tian J. H. Pan P. Y. Zald P. Li C. Deng C. . (2008). Docking of axonal mitochondria by syntaphilin controls their mobility and affects short-term facilitation. Cell 132, 137–148. 10.1016/j.cell.2007.11.024 18191227 PMC2259239

[B104] Kannarkat G. T. Boss J. M. Tansey M. G. (2013). The role of innate and adaptive immunity in Parkinson's disease. J. Parkinsons. Dis. 3, 493–514. 10.3233/JPD-130250 24275605 PMC4102262

[B105] Karikari A. A. McFleder R. L. Ribechini E. Blum R. Bruttel V. Knorr S. . (2022). Neurodegeneration by α-synuclein-specific T cells in AAV-A53T-α-synuclein Parkinson's disease mice. Brain Behav. Immun. 101, 194–210. 10.1016/j.bbi.2022.01.007 35032575

[B106] Kempuraj D. Khan M. M. Thangavel R. Xiong Z. Yang E. Zaheer A. (2013). Glia maturation factor induces interleukin-33 release from astrocytes: implications for neurodegenerative diseases. J. Neuroimmune Pharmacol. 8, 643–650. 10.1007/s11481-013-9439-7 23397250 PMC3660415

[B107] Kempuraj D. Thangavel R. Fattal R. Pattani S. Yang E. Zaheer S. . (2016). Mast cells release chemokine CCL2 in response to parkinsonian toxin 1-methyl-4-phenyl-pyridinium (MPP(+)). Neurochem. Res. 41, 1042–1049. 10.1007/s11064-015-1790-z 26646004 PMC4834226

[B108] Kempuraj D. Thangavel R. Selvakumar G. P. Ahmed M. E. Zaheer S. Raikwar S. P. . (2019). Mast Cell proteases activate astrocytes and glia-neurons and release interleukin-33 by activating p38 and ERK1/2 MAPKs and NF-κB. Mol. Neurobiol. 56, 1681–1693. 10.1007/s12035-018-1177-7 29916143 PMC6298852

[B109] Kempuraj D. Thangavel R. Yang E. Pattani S. Zaheer S. Santillan D. A. . (2015). Dopaminergic toxin 1-methyl-4-phenylpyridinium, proteins α-synuclein and glia maturation factor activate mast cells and release inflammatory mediators. PLoS ONE 10, e0135776. 10.1371/journal.pone.0135776 26275153 PMC4537263

[B110] Kilpeläinen T. Julku U. H. Svarcbahs R. Myöhänen T. T. (2019). Behavioural and dopaminergic changes in double mutated human A30P^*^A53T alpha-synuclein transgenic mouse model of Parkinson's disease. Sci. Rep. 9, 17382. 10.1038/s41598-019-54034-z PMC6874660 31758049

[B111] Kim B. W. Jeong K. H. Kim J. H. Jin M. Kim J. H. Lee M. G. . (2016). Pathogenic upregulation of glial lipocalin-2 in the parkinsonian dopaminergic system. J. Neurosci. 36, 5608–5622. 10.1523/JNEUROSCI.4261-15.2016 27194339 PMC6601774

[B112] Kim C. Ho D. H. Suk J. E. You S. Michael S. Kang J. . (2013). Neuron-released oligomeric α-synuclein is an endogenous agonist of TLR2 for paracrine activation of microglia. Nat. Commun. 4, 1562. 10.1038/ncomms2534 PMC4089961 23463005

[B113] Kim J. Fiesel F. C. Belmonte K. C. Hudec R. Wang W.-X. Kim C. . (2016). miR-27a and miR-27b regulate autophagic clearance of damaged mitochondria by targeting PTEN-induced putative kinase 1 (PINK1). Mol. Neurodegener. 11, 55. 10.1186/s13024-016-0121-4 PMC4960690 27456084

[B114] Klegeris A.. (2021). Regulation of neuroimmune processes by damage- and resolution-associated molecular patterns. Neural Regen. Res. 16, 423–429. 10.4103/1673-5374.293134 32985460 PMC7996015

[B115] Klegeris A. Giasson B. I. Zhang H. Maguire J. Pelech S. McGeer P. L. (2006). Alpha-synuclein and its disease-causing mutants induce ICAM-1 and IL-6 in human astrocytes and astrocytoma cells. FASEB J. 20, 2000–2008. 10.1096/fj.06-6183com 17012252

[B116] Kokhan V. Afanasyeva M. Van'Kin G. (2012). α-Synuclein knockout mice have cognitive impairments. Behav. Brain Res. 231, 226–230. 10.1016/j.bbr.2012.03.026 22469626

[B117] Kumar R. Agarwal A. K. Seth P. K. (1995). Free radical-generated neurotoxicity of 6-hydroxydopamine. J. Neurochem. 64, 1703–1707. 10.1046/j.1471-4159.1995.64041703.x 7891098

[B118] Kurkowska-Jastrzebska I. Wrońska A. Kohutnicka M. Członkowski A. Członkowska A. (1999). MHC class II positive microglia and lymphocytic infiltration are present in the substantia nigra and striatum in mouse model of Parkinson's disease. Acta Neurobiol. Exp. 59, 1–8. 10230070 10.55782/ane-1999-1289

[B119] La Vitola P. Balducci C. Baroni M. Artioli L. Santamaria G. Castiglioni M. . (2021). Peripheral inflammation exacerbates α-synuclein toxicity and neuropathology in Parkinson's models. Neuropathol. Appl. Neurobiol. 47, 43–60. 10.1111/nan.12644 32696999

[B120] Labzin L. I. Heneka M. T. Latz E. (2018). Innate immunity and neurodegeneration. Annu. Rev. Med. 69, 437–449. 10.1146/annurev-med-050715-104343 29106805

[B121] Lai T. T. Kim Y. J. Nguyen P. T. Koh Y. H. Nguyen T. T. Ma H. I. . (2021). Temporal evolution of inflammation and neurodegeneration with alpha-synuclein propagation in Parkinson's disease mouse model. Front. Integr. Neurosci. 15, 715190. 10.3389/fnint.2021.715190 PMC8523784 34675786

[B122] Lawrence T.. (2009). The nuclear factor NF-kappaB pathway in inflammation. Cold Spring Harb. Perspect. Biol. 1, a001651. 10.1101/cshperspect.a001651 PMC2882124 20457564

[B123] Lee H. J. Bae E. J. Lee S. J. (2014). Extracellular α-synuclein-a novel and crucial factor in Lewy body diseases. Nat. Rev. Neurol. 10, 92–98. 10.1038/nrneurol.2013.275 24468877

[B124] Lee K. W. Zhao X. Im J. Y. Grosso H. Jang W. H. Chan T. W. . (2012). Apoptosis signal-regulating kinase 1 mediates MPTP toxicity and regulates glial activation. PLoS ONE 7, e29935. 10.1371/journal.pone.0029935 22253830 PMC3254627

[B125] Leitner G. R. Wenzel T. J. Marshall N. Gates E. J. Klegeris A. (2019). Targeting toll-like receptor 4 to modulate neuroinflammation in central nervous system disorders. Expert Opin. Ther. Targets 23, 865–882. 10.1080/14728222.2019.1676416 31580163

[B126] Li H. Yang J. Wang Y. Liu Q. Cheng J. Wang F. (2019). Neuroprotective effects of increasing levels of HSP70 against neuroinflammation in Parkinson's disease model by inhibition of NF-κB and STAT3. Life Sci. 234, 116747. 10.1016/j.lfs.2019.116747 31408661

[B127] Li L. Nadanaciva S. Berger Z. Shen W. Paumier K. Schwartz J. . (2013). Human A53T α-synuclein causes reversible deficits in mitochondrial function and dynamics in primary mouse cortical neurons. PLoS ONE 8, e85815. 10.1371/journal.pone.0085815 24392030 PMC3877382

[B128] Li Y. Niu M. Zhao A. Kang W. Chen Z. Luo N. . (2019). CXCL12 is involved in α-synuclein-triggered neuroinflammation of Parkinson's disease. J. Neuroinflammation 16, 263. 10.1186/s12974-019-1646-6 31831012 PMC6909602

[B129] Liberatore G. T. Jackson-Lewis V. Vukosavic S. Mandir A. S. Vila M. McAuliffe W. G. . (1999). Inducible nitric oxide synthase stimulates dopaminergic neurodegeneration in the MPTP model of Parkinson disease. Nat. Med. 5, 1403–1409. 10.1038/70978 10581083

[B130] Lim R. Zaheer A. Khosravi H. Freeman J. H. Jr Halverson H. E. Wemmie J. A. Yang B. (2004). Impaired motor performance and learning in glia maturation factor-knockout mice. Brain Res. 1024, 225–232. 10.1016/j.brainres.2004.08.003 15451385

[B131] Lin C.-Y. Tsai C.-W. (2017). Carnosic acid attenuates 6-hydroxydopamine-induced neurotoxicity in SH-SY5Y cells by inducing autophagy through an enhanced interaction of parkin and Beclin1. Mol. Neurobiol. 54, 2813–2822. 10.1007/s12035-016-9873-7 27013469

[B132] Lopes F. M. Bristot I. J. da Motta L. L. Parsons R. B. Klamt F. (2017a). Mimicking Parkinson's disease in a dish: merits and pitfalls of the most commonly used dopaminergic *in vitro* models. Neuromol. Med. 19, 241–255. 10.1007/s12017-017-8454-x 28721669

[B133] Lopes F. M. da Motta L. L. De Bastiani M. A. Pfaffenseller B. Aguiar B. W. de Souza L. F. . (2017b). RA differentiation enhances dopaminergic features, changes redox parameters, and increases dopamine transporter dependency in 6-hydroxydopamine-induced neurotoxicity in SH-SY5Y cells. Neurotox. Res. 31, 545–559. 10.1007/s12640-016-9699-0 28155214

[B134] Lopes F. M. Schröder R. da Frota Júnior M. L. C. Zanotto-Filho A. Müller C. B. Pires A. S. . (2010). Comparison between proliferative and neuron-like SH-SY5Y cells as an *in vitro* model for Parkinson disease studies. Brain Res. 1337, 85–94. 10.1016/j.brainres.2010.03.102 20380819

[B135] Lunderius-Andersson C. Enoksson M. Nilsson G. (2012). Mast cells respond to cell injury through the recognition of IL-33. Front. Immunol. 3, 82. 10.3389/fimmu.2012.00082 PMC3342375 22566963

[B136] Lyman M. Lloyd D. G. Ji X. Vizcaychipi M. P. Ma D. (2014). Neuroinflammation: the role and consequences. Neurosci Res. 79, 1–12. 10.1016/j.neures.2013.10.004 24144733

[B137] Magen I. Chesselet M.-F. (2011). Mouse models of cognitive deficits due to alpha-synuclein pathology. J. Parkinsons Dis. 1, 217–227. 10.3233/JPD-2011-11043 23939303

[B138] Main B. S. Zhang M. Brody K. M. Ayton S. Frugier T. Steer D. . (2016). Type-1 interferons contribute to the neuroinflammatory response and disease progression of the MPTP mouse model of Parkinson's disease. Glia. 64, 1590–1604. 10.1002/glia.23028 27404846

[B139] Main B. S. Zhang M. Brody K. M. Kirby F. J. Crack P. J. Taylor J. M. (2017). Type-I interferons mediate the neuroinflammatory response and neurotoxicity induced by rotenone. J. Neurochem. 141, 75–85. 10.1111/jnc.13940 28029694

[B140] Mao J. Gao H. Bai W. Zeng H. Ren Y. Liu Y. . (2021). Lipoic acid alleviates LPS-evoked PC12 cell damage by targeting p53 and inactivating the NF-κB pathway. Acta Neurobiol. Exp. 81, 375–385. 10.21307/ane-2021-037 35014986

[B141] Markowitz J. Carson W. E. (2013). Review of S100A9 biology and its role in cancer. Biochim. Biophys. Acta. 1835, 100–109. 10.1016/j.bbcan.2012.10.003 23123827 PMC3670606

[B142] Marques O. Outeiro T. F. (2012). Alpha-synuclein: from secretion to dysfunction and death. Cell Death Dis. 3, e350–e350. 10.1038/cddis.2012.94 22825468 PMC3406593

[B143] McGeer E. G. McGeer P. L. (2007). The role of anti-inflammatory agents in Parkinson's disease. CNS Drugs 21, 789–797. 10.2165/00023210-200721100-00001 17850169

[B144] Mendez-Gomez H. R. Singh J. Meyers C. Chen W. Gorbatyuk O. S. Muzyczka N. (2018). The lipase activity of phospholipase D2 is responsible for nigral neurodegeneration in a rat model of Parkinson's disease. Neuroscience 377, 174–183. 10.1016/j.neuroscience.2018.02.047 29526688 PMC5882525

[B145] Miklossy J. Doudet D. D. Schwab C. Yu S. McGeer E. G. McGeer P. L. (2006). Role of ICAM-1 in persisting inflammation in Parkinson disease and MPTP monkeys. Exp. Neurol. 197, 275–283. 10.1016/j.expneurol.2005.10.034 16336966

[B146] Min B. Chung K. C. (2018). New insight into transglutaminase 2 and link to neurodegenerative diseases. BMB Rep. 51, 5–13. 10.5483/BMBRep.2018.51.1.227 29187283 PMC5796628

[B147] Min B. Kwon Y. C. Choe K. M. Chung K. C. (2015). PINK1 phosphorylates transglutaminase 2 and blocks its proteasomal degradation. J. Neurosci. Res. 93, 722–735. 10.1002/jnr.23535 25557247

[B148] Mitra S. Chakrabarti N. Bhattacharyya A. (2011). Differential regional expression patterns of α-synuclein, TNF-α, and IL-1β; and variable status of dopaminergic neurotoxicity in mouse brain after paraquat treatment. J. Neuroinflammation 8, 1–22. 10.1186/1742-2094-8-163 22112368 PMC3247140

[B149] Mohamadkhani A.. (2018). Gut microbiota and fecal metabolome perturbation in children with autism spectrum disorder. Middle East J Dig Dis. 10, 205–212. 10.15171/mejdd.2018.112 31049167 PMC6488507

[B150] Mohamadkhani A. Sotoudeh M. Bowden S. Poustchi H. Jazii F. R. Sayehmiri K. . (2009). Downregulation of HLA class II molecules by G1896A pre-core mutation in chronic hepatitis B virus infection. Viral Immunol. 22, 295–300. 10.1089/vim.2009.0031 19811086

[B151] Molteni M. Rossetti C. (2017). Neurodegenerative diseases: the immunological perspective. J. Neuroimmunol. 313, 109–115. 10.1016/j.jneuroim.2017.11.002 29153601

[B152] Molteni R. Macchi F. Zecchillo C. Dell'Agli M. Colombo E. Calabrese F. . (2013). Modulation of the inflammatory response in rats chronically treated with the antidepressant agomelatine. Euro. Neuropsychopharmacol. 23, 1645–1655. 10.1016/j.euroneuro.2013.03.008 23622958

[B153] Morales-Garcia J. A. Alonso-Gil S. Gil C. Martinez A. Santos A. Perez-Castillo A. (2015). Phosphodiesterase 7 inhibition induces dopaminergic neurogenesis in hemiparkinsonian rats. Stem Cells Transl. Med. 4, 564–575. 10.5966/sctm.2014-0277 25925836 PMC4449102

[B154] Morales-Garcia J. A. Alonso-Gil S. Santos Á. Perez-Castillo A. (2020). Phosphodiesterase 7 regulation in cellular and rodent models of Parkinson's disease. Mol. Neurobiol. 57, 806–822. 10.1007/s12035-019-01745-z 31473904

[B155] Morales-Garcia J. A. Echeverry-Alzate V. Alonso-Gil S. Sanz-SanCristobal M. Lopez-Moreno J. A. Gil C. . (2017). Phosphodiesterase7 inhibition activates adult neurogenesis in hippocampus and subventricular zone *in vitro* and in vivo. Stem Cells 35, 458–472. 10.1002/stem.2480 27538853

[B156] Morales-Garcia J. A. Redondo M. Alonso-Gil S. Gil C. Perez C. Martinez A. . (2011). Phosphodiesterase 7 inhibition preserves dopaminergic neurons in cellular and rodent models of Parkinson disease. PLoS ONE 6, e17240. 10.1371/journal.pone.0017240 21390306 PMC3044733

[B157] Mukai K. Tsai M. Saito H. Galli S. J. (2018). Mast cells as sources of cytokines, chemokines growth factors. Immunol. Rev. 282, 121–150. 10.1111/imr.12634 29431212 PMC5813811

[B158] Murray R. Z. Kay J. G. Sangermani D. G. Stow J. L. (2005). A role for the phagosome in cytokine secretion. Science 310, 1492–1495. 10.1126/science.1120225 16282525

[B159] Nalls M. A. Blauwendraat C. Vallerga C. L. Heilbron K. Bandres-Ciga S. Chang D. . (2019). Identification of novel risk loci, causal insights, and heritable risk for Parkinson's disease: a meta-analysis of genome-wide association studies. Lancet Neurol. 18, 1091–1102. 10.1016/S1474-4422(19)30320-5 31701892 PMC8422160

[B160] Neal M. L. Boyle A. M. Budge K. M. Safadi F. F. Richardson J. R. (2018). The glycoprotein GPNMB attenuates astrocyte inflammatory responses through the CD44 receptor. J. Neuroinflammation 15, 73. 10.1186/s12974-018-1100-1 29519253 PMC5842560

[B161] Nikodemova M. Watters J. J. (2011). Outbred ICR/CD1 mice display more severe neuroinflammation mediated by microglial TLR4/CD14 activation than inbred C57Bl/6 mice. Neuroscience 190, 67–74. 10.1016/j.neuroscience.2011.06.006 21683771 PMC3156380

[B162] Nishibori M. Mori S. Takahashi H. K. (2019). Anti-HMGB1 monoclonal antibody therapy for a wide range of CNS and PNS diseases. J. Pharmacol. Sci. 140, 94–101. 10.1016/j.jphs.2019.04.006 31105025

[B163] Noh H. Jeon J. Seo H. (2014). Systemic injection of LPS induces region-specific neuroinflammation and mitochondrial dysfunction in normal mouse brain. Neurochem. Int. 69, 35–40. 10.1016/j.neuint.2014.02.008 24607701

[B164] Olesen M. N. Christiansen J. R. Petersen S. V. Jensen P. H. Paslawski W. Romero-Ramos M. . (2018). CD4 T cells react to local increase of α-synuclein in a pathology-associated variant-dependent manner and modify brain microglia in absence of brain pathology. Heliyon 4, e00513. 10.1016/j.heliyon.2018.e00513 29560431 PMC5857520

[B165] Olson K. E. Gendelman H. E. (2016). Immunomodulation as a neuroprotective and therapeutic strategy for Parkinson's disease. Curr. Opin. Pharmacol. 26, 87–95. 10.1016/j.coph.2015.10.006 26571205 PMC4716884

[B166] Oskvig D. B. Elkahloun A. G. Johnson K. R. Phillips T. M. Herkenham M. (2012). Maternal immune activation by LPS selectively alters specific gene expression profiles of interneuron migration and oxidative stress in the fetus without triggering a fetal immune response. Brain Behav. Immun. 26, 623–634. 10.1016/j.bbi.2012.01.015 22310921 PMC3285385

[B167] Panicker N. Sarkar S. Harischandra D. S. Neal M. Kam T. I. Jin H. . (2019). Fyn kinase regulates misfolded α-synuclein uptake and NLRP3 inflammasome activation in microglia. J. Exp. Med. 216, 1411–1430. 10.1084/jem.20182191 31036561 PMC6547864

[B168] Parihar M. S. Parihar A. Fujita M. Hashimoto M. Ghafourifar P. (2009). Alpha-synuclein overexpression and aggregation exacerbates impairment of mitochondrial functions by augmenting oxidative stress in human neuroblastoma cells. Int. J. Biochem. Cell Biol. 41, 2015–2024. 10.1016/j.biocel.2009.05.008 19460457

[B169] Park D. R. Thomsen A. R. Frevert C. W. Pham U. Skerrett S. J. Kiener P. A. . (2003). Fas (CD95) induces proinflammatory cytokine responses by human monocytes and monocyte-derived macrophages. J. Immunol. 170, 6209–6216. 10.4049/jimmunol.170.12.6209 12794152

[B170] Park J. Lee J. W. Cooper S. C. Broxmeyer H. E. Cannon J. R. Kim C. H. (2017). Parkinson disease-associated LRRK2 G2019S transgene disrupts marrow myelopoiesis and peripheral Th17 response. J. Leukoc. Biol. 102, 1093–1102. 10.1189/jlb.1A0417-147RR 28751472 PMC5597519

[B171] Pathak D. Sepp K. J. Hollenbeck P. J. (2010). Evidence that myosin activity opposes microtubule-based axonal transport of mitochondria. J. Neurosci. 30, 8984–8992. 10.1523/JNEUROSCI.1621-10.2010 20592219 PMC2904968

[B172] Paumier K. L. Luk K. C. Manfredsson F. P. Kanaan N. M. Lipton J. W. Collier T. J. . (2015). Intrastriatal injection of pre-formed mouse α-synuclein fibrils into rats triggers α-synuclein pathology and bilateral nigrostriatal degeneration. Neurobiol. Dis. 82, 185–199. 10.1016/j.nbd.2015.06.003 26093169 PMC4640952

[B173] Perese D. Ulman J. Viola J. Ewing S. Bankiewicz K. (1989). A 6-hydroxydopamine-induced selective parkinsonian rat model. Brain Res. 494, 285–293. 10.1016/0006-8993(89)90597-0 2528389

[B174] Perumal A. Gopal V. Tordzro W. Cooper T. Cadet J. (1992). Vitamin E attenuates the toxic effects of 6-hydroxydopamine on free radical scavenging systems in rat brain. Brain Res. Bull. 29, 699–701. 10.1016/0361-9230(92)90142-K 1422867

[B175] Pourasgari M. Mohamadkhani A. (2020). Heritability for stroke: essential for taking family history. Caspian J. Intern. Med. 11, 237–243. 10.22088/cjim.11.3.237 32874429 PMC7442467

[B176] Przedborski S. Jackson-Lewis V. Djaldetti R. Liberatore G. Vila M. Vukosavic S. . (2000). The parkinsonian toxin MPTP: action and mechanism. Restor. Neurol. Neurosci. 16, 135–142. 12671216

[B177] Przedborski S. Tieu K. Perier C. Vila M. (2004). MPTP as a mitochondrial neurotoxic model of Parkinson's Disease. J. Bioenerg. Biomembr. 36, 375–379. 10.1023/B:JOBB.0000041771.66775.d5 15377875

[B178] Przedbroski S. Leviver M. Jiang H. Ferreira M. Jackson-Lewis V. Donaldson D. . (1995). Dose-dependent lesions of the dopaminergic nigrostriatal pathway induced by instrastriatal injection of 6-hydroxydopamine. Neuroscience 67, 631–647. 10.1016/0306-4522(95)00066-R 7675192

[B179] Purisai M. G. McCormack A. L. Cumine S. Li J. Isla M. Z. Di Monte D. A. (2007). Microglial activation as a priming event leading to paraquat-induced dopaminergic cell degeneration. Neurobiol. Dis. 25, 392–400. 10.1016/j.nbd.2006.10.008 17166727 PMC2001246

[B180] Qiao Y. Wang P. Qi J. Zhang L. Gao C. (2012). TLR-induced NF-κB activation regulates NLRP3 expression in murine macrophages. FEBS Lett. 586, 1022–1026. 10.1016/j.febslet.2012.02.045 22569257

[B181] Qin L. Wu X. Block M. L. Liu Y. Breese G. R. Hong J. S. . (2007). Systemic LPS causes chronic neuroinflammation and progressive neurodegeneration. Glia 55, 453–462. 10.1002/glia.20467 17203472 PMC2871685

[B182] Qin X. Y. Zhang S. P. Cao C. Loh Y. P. Cheng Y. (2016). Aberrations in peripheral inflammatory cytokine levels in Parkinson disease: a systematic review and meta-analysis. JAMA Neurol. 73, 1316–1324. 10.1001/jamaneurol.2016.2742 27668667

[B183] Ransohoff R. M.. (2016). How neuroinflammation contributes to neurodegeneration. Science 353, 7777–83. 10.1126/science.aag2590 27540165

[B184] Ransohoff R. M. Brown M. A. (2012). Innate immunity in the central nervous system. J. Clin. Invest. 122, 1164–1171. 10.1172/JCI58644 22466658 PMC3314450

[B185] Reynolds A. D. Banerjee R. Liu J. Gendelman H. E. Mosley R. L. (2007). Neuroprotective activities of CD4+CD25+ regulatory T cells in an animal model of Parkinson's disease. J. Leukoc. Biol. 82, 1083–1094. 10.1189/jlb.0507296 17675560

[B186] Rodrigues M. C. Sanberg P. R. Cruz L. E. Garbuzova-Davis S. (2014). The innate and adaptive immunological aspects in neurodegenerative diseases. J. Neuroimmunol. 269, 1–8. 10.1016/j.jneuroim.2013.09.020 24161471

[B187] Sachs C. Jonsson G. (1975). Mechanisms of action of 6-hydroxydopamine. Biochem. Pharmacol. 24, 1–8. 10.1016/0006-2952(75)90304-4 1092302

[B188] Sandhu J. K. Kulka M. (2021). Decoding mast cell-microglia communication in neurodegenerative diseases. Int. J. Mol. Sci. 22, 1093. 10.3390/ijms22031093 PMC7865982 33499208

[B189] Sarkar S. Dammer E. B. Malovic E. Olsen A. L. Raza S. A. Gao T. . (2020a). Molecular signatures of neuroinflammation induced by αsynuclein aggregates in microglial cells. Front. Immunol. 11, 33. 10.3389/fimmu.2020.00033 PMC7006296 32082315

[B190] Sarkar S. Nguyen H. M. Malovic E. Luo J. Langley M. Palanisamy B. N. . (2020b). Kv1.3 modulates neuroinflammation and neurodegeneration in Parkinson's disease. J. Clin. Invest. 130, 4195–4212. 10.1172/JCI136174 32597830 PMC7410064

[B191] Schildknecht S. Pöltl D. Nagel D. M. Matt F. Scholz D. Lotharius J. . (2009). Requirement of a dopaminergic neuronal phenotype for toxicity of low concentrations of 1-methyl-4-phenylpyridinium to human cells. Toxicol. Appl. Pharmacol. 241, 23–35. 10.1016/j.taap.2009.07.027 19647008

[B192] Scholz D. Pöltl D. Genewsky A. Weng M. Waldmann T. Schildknecht S. . (2011). Rapid, complete and large-scale generation of post-mitotic neurons from the human LUHMES cell line. J. Neurochem. 119, 957–971. 10.1111/j.1471-4159.2011.07255.x 21434924

[B193] Schwarting R. Huston J. (1996). Unilateral 6-hydroxydopamine lesions of meso-striatal dopamine neurons and their physiological sequelae. Prog. Neurobiol. 49, 215–266. 10.1016/S0301-0082(96)00015-9 8878304

[B194] Senior S. L. Ninkina N. Deacon R. Bannerman D. Buchman V. L. Cragg S. J. . (2008). Increased striatal dopamine release and hyperdopaminergic-like behaviour in mice lacking both alpha-synuclein and gamma-synuclein. Eur J Neurosci. 27, 947–957. 10.1111/j.1460-9568.2008.06055.x 18333965 PMC3145106

[B195] Servier (2022). Smart News. Servier. Available online at: https://smart.servier.com/

[B196] Shao Q.-H. Chen Y. Li F.-F, Wang, S. Zhang X.-L. Yuan Y.-H. . (2019). TLR4 deficiency has a protective effect in the MPTP/probenecid mouse model of Parkinson's disease. Acta Pharmacol. Sin. 40, 1503–1512. 10.1038/s41401-019-0280-2 31388087 PMC7471440

[B197] Shin W. H. Jeon M. T. Leem E. Won S. Y. Jeong K. H. Park S. J. . (2015). Induction of microglial toll-like receptor 4 by prothrombin kringle-2: a potential pathogenic mechanism in Parkinson's disease. Sci. Rep. 5, 14764. 10.1038/srep14764 PMC4594003 26440368

[B198] Simón-Sánchez J. Schulte C. Bras J. M. Sharma M. Gibbs J. R. Berg D. . (2009). Genome-wide association study reveals genetic risk underlying Parkinson's disease. Nat. Genet. 41, 1308–1312. 10.1038/ng.487 19915575 PMC2787725

[B199] Sims G. P. Rowe D. C. Rietdijk S. T. Herbst R. Coyle A. J. (2010). HMGB1 and RAGE in inflammation and cancer. Annu. Rev. Immunol. 28, 367–388. 10.1146/annurev.immunol.021908.132603 20192808

[B200] Singh A. Tripathi P. Singh S. (2021). Neuroinflammatory responses in Parkinson's disease: relevance of ibuprofen in therapeutics. Inflammopharmacology 29, 5–14. 10.1007/s10787-020-00764-w 33052479

[B201] Singh S. S. Rai S. N. Birla H. Zahra W. Rathore A. S. Singh S. P. (2020). NF-κB-mediated neuroinflammation in Parkinson's disease and potential therapeutic effect of polyphenols. Neurotox. Res. 37, 491–507. 10.1007/s12640-019-00147-2 31823227

[B202] Slanzi A. Iannoto G. Rossi B. Zenaro E. Constantin G. (2020). *In vitro* models of neurodegenerative diseases. Front. Cell Dev. Biol. 8, 328. 10.3389/fcell.2020.00328 PMC7247860 32528949

[B203] Soto-Otero R. Méndez-Álvarez E. Hermida-Ameijeiras Á. Muñoz-Patiño A. M. Labandeira-Garcia J. L. (2000). Autoxidation and neurotoxicity of 6-hydroxydopamine in the presence of some antioxidants: potential implication in relation to the pathogenesis of Parkinson's disease. J. Neurochem. 74, 1605–1612. 10.1046/j.1471-4159.2000.0741605.x 10737618

[B204] Specht C. G. Schoepfer R. (2004). Deletion of multimerin-1 in α-synuclein-deficient mice. Genomics 83, 1176–1178. 10.1016/j.ygeno.2003.12.014 15177571

[B205] Srikrishna G.. (2012). S100A8 and S100A9: new insights into their roles in malignancy. J. Innate Immun. 4, 31–40. 10.1159/000330095 21912088 PMC3250655

[B206] Steiner J. A. Quansah E. Brundin P. (2018). The concept of alpha-synuclein as a prion-like protein: ten years after. Cell Tissue Res. 373, 161–173. 10.1007/s00441-018-2814-1 29480459 PMC6541204

[B207] Stojkovska I. Wagner B. M. Morrison B. E. (2015). Parkinson's disease and enhanced inflammatory response. Exp. Biol. Med. 240, 1387–1395. 10.1177/1535370215576313 25769314 PMC4935292

[B208] Storelli E. Cassina N. Rasini E. Marino F. Cosentino M. (2019). Do Th17 lymphocytes and IL-17 contribute to Parkinson's disease? A systematic review of available evidence. Front. Neurol. 10, 13. 10.3389/fneur.2019.00013 PMC6353825 30733703

[B209] Subbarayan M. S. Hudson C. Moss L. D. Nash K. R. Bickford P. C. (2020). T cell infiltration and upregulation of MHCII in microglia leads to accelerated neuronal loss in an α-synuclein rat model of Parkinson's disease. J. Neuroinflammation 17, 242. 10.1186/s12974-020-01911-4 32799878 PMC7429710

[B210] Sulzer D. Alcalay R. N. Garretti F. Cote L. Kanter E. Agin-Liebes J. . (2017). T cells from patients with Parkinson's disease recognize α-synuclein peptides. Nature 546, 656–661. 10.1038/nature22815 28636593 PMC5626019

[B211] Sun Y. Wen Y. Wang L. Wen L. You W. Wei S. . (2021). Therapeutic opportunities of interleukin-33 in the central nervous system. Front. Immunol. 12, 654626. 10.3389/fimmu.2021.654626 PMC8165230 34079543

[B212] Tansey M. G. Goldberg M. S. (2010). Neuroinflammation in Parkinson's disease: its role in neuronal death and implications for therapeutic intervention. Neurobiol. Dis. 37, 510–518. 10.1016/j.nbd.2009.11.004 19913097 PMC2823829

[B213] Taracanova A. Alevizos M. Karagkouni A. Weng Z. Norwitz E. Conti P. . (2017). SP and IL-33 together markedly enhance TNF synthesis and secretion from human mast cells mediated by the interaction of their receptors. Proc. Natl. Acad. Sci. U.S.A. 114, E4002–e4009. 10.1073/pnas.1524845114 28461492 PMC5441798

[B214] Thakur P. Breger L. S. Lundblad M. Wan O. W. Mattsson B. Luk K. C. . (2017). Modeling Parkinson's disease pathology by combination of fibril seeds and α-synuclein overexpression in the rat brain. Proc. Natl. Acad. Sci. U.S.A. 114, E8284–e8293. 10.1073/pnas.1710442114 28900002 PMC5625925

[B215] Theodore S. Cao S. McLean P. J. Standaert D. G. (2008). Targeted overexpression of human alpha-synuclein triggers microglial activation and an adaptive immune response in a mouse model of Parkinson disease. J. Neuropathol. Exp. Neurol. 67, 1149–1158. 10.1097/NEN.0b013e31818e5e99 19018246 PMC2753200

[B216] Thomas M. Saldanha M. Mistry R. Dexter D. Ramsden D. Parsons R. (2013). Nicotinamide N-methyltransferase expression in SH-SY5Y neuroblastoma and N27 mesencephalic neurones induces changes in cell morphology via ephrin-B2 and Akt signalling. Cell Death Dis. 4, e669–e669. 10.1038/cddis.2013.200 23764850 PMC3702289

[B217] Titze-de-Almeida R. Titze-de-Almeida S. S. (2018). miR-7 replacement therapy in Parkinson's Disease. Curr. Gene Ther. 18, 143–153. 10.2174/1566523218666180430121323 29714132

[B218] Tobon-Velasco C. J. Cuevas E. Torres-Ramos M. A. (2014). Receptor for AGEs (RAGE) as mediator of NF-kB pathway activation in neuroinflammation and oxidative stress. CNS Neurol. Disord. Drug Targ. 13, 1615–1626. 10.2174/1871527313666140806144831 25106630

[B219] Tore F. Tuncel N. (2009). Mast cells: target and source of neuropeptides. Curr. Pharm. Des. 15, 3433–3445. 10.2174/138161209789105036 19860689

[B220] Trudler D. Nash Y. Frenkel D. (2015). New insights on Parkinson's disease genes: the link between mitochondria impairment and neuroinflammation. J. Neural Transm. 122, 1409–1419. 10.1007/s00702-015-1399-z 25894287

[B221] Trudler D. Nazor K. L. Eisele Y. S. Grabauskas T. Dolatabadi N. Parker J. . (2021). Soluble α-synuclein-antibody complexes activate the NLRP3 inflammasome in hiPSC-derived microglia. Proc. Natl. Acad. Sci. U.S.A. 118, e2025847118. 10.1073/pnas.2025847118 PMC8054017 33833060

[B222] Tweedie D. Sambamurti K. Greig N. H. (2007). TNF-α inhibition as a treatment strategy for neurodegenerative disorders: new drug candidates and targets. Curr. Alzheimer Res. 4, 378–385. 10.2174/156720507781788873 17908040

[B223] Uematsu S. Matsumoto M. Takeda K. Akira S. (2002). Lipopolysaccharide-dependent prostaglandin E(2) production is regulated by the glutathione-dependent prostaglandin E(2) synthase gene induced by the Toll-like receptor 4/MyD88/NF-IL6 pathway. J. Immunol. 168, 5811–5816. 10.4049/jimmunol.168.11.5811 12023384

[B224] Ungerstedt U.. (1968). 6-Hydroxy-dopamine induced degeneration of central monoamine neurons. Eur. J. Pharmacol. 5, 107–110. 10.1016/0014-2999(68)90164-7 5718510

[B225] Verhaar R. Drukarch B. Bol J. G. Jongenelen C. A. Musters R. J. Wilhelmus M. M. (2012). Increase in endoplasmic reticulum-associated tissue transglutaminase and enzymatic activation in a cellular model of Parkinson's disease. Neurobiol. Dis. 45, 839–850. 10.1016/j.nbd.2011.10.012 22051113

[B226] Verhaar R. Jongenelen C. A. Gerard M. Baekelandt V. Van Dam A. M. Wilhelmus M. M. . (2011). Blockade of enzyme activity inhibits tissue transglutaminase-mediated transamidation of α-synuclein in a cellular model of Parkinson's disease. Neurochem. Int. 58, 785–793. 10.1016/j.neuint.2011.03.004 21440023

[B227] von Euler Chelpin M. Vorup-Jensen T. (2017). Targets and mechanisms in prevention of Parkinson's disease through immunomodulatory treatments. Scand. J. Immunol. 85, 321–330. 10.1111/sji.12542 28231624

[B228] Wang S. Yuan Y.-H. Chen N.-H. Wang H.-B. (2019). The mechanisms of NLRP3 inflammasome/pyroptosis activation and their role in Parkinson's disease. Int. Immunopharmacol. 67, 458–464. 10.1016/j.intimp.2018.12.019 30594776

[B229] Wang X. Schwarz T. L. (2009). The mechanism of Ca2+ -dependent regulation of kinesin-mediated mitochondrial motility. Cell 136, 163–174. 10.1016/j.cell.2008.11.046 19135897 PMC2768392

[B230] Wang Y. Li L. Hou C. Lai Y. Long J. Liu J. . (2020). SNARE-mediated membrane fusion in autophagy. Semi. Cell Dev. Biol. 60, 97–104. 10.1016/j.semcdb.2016.07.009 27422330 PMC5161566

[B231] Wei L. Ding L. Mo M.-,s, Lei, M. Zhang L. Chen K. Xu P. (2015). Wnt3a protects SH-SY5Y cells against 6-hydroxydopamine toxicity by restoration of mitochondria function. Transl. Neurodegener. 4, 1–8. 10.1186/s40035-015-0033-1 26085927 PMC4470059

[B232] Wenzel T. J. Kwong E. Bajwa E. Klegeris A. (2020). Resolution-associated molecular patterns (RAMPs) as endogenous regulators of glia functions in neuroinflammatory disease. CNS Neurol. Disord. Drug Targets. 19, 483–494. 10.2174/1871527319666200702143719 32614758

[B233] Wilhelmus M. M. Verhaar R. Andringa G. Bol J. G. Cras P. Shan L. . (2011). Presence of tissue transglutaminase in granular endoplasmic reticulum is characteristic of melanized neurons in Parkinson's disease brain. Brain Pathol. 21, 130–139. 10.1111/j.1750-3639.2010.00429.x 20731657 PMC8094245

[B234] Williams G. P. Schonhoff A. M. Jurkuvenaite A. Gallups N. J. Standaert D. G. Harms A. S. (2021). CD4 T cells mediate brain inflammation and neurodegeneration in a mouse model of Parkinson's disease. Brain 144, 2047–2059. 10.1093/brain/awab103 33704423 PMC8370411

[B235] Wyss-Coray T. Mucke L. (2002). Inflammation in neurodegenerative disease—a double-edged sword. Neuron 35, 419–432. 10.1016/S0896-6273(02)00794-8 12165466

[B236] Xiao H. X. Song B. Li Q. Shao Y. M. Zhang Y. B. Chang X. L. . (2022). Paraquat mediates BV-2 microglia activation by raising intracellular ROS and inhibiting Akt1 phosphorylation. Toxicol. Lett. 355, 116–126. 10.1016/j.toxlet.2021.11.017 34863858

[B237] Xicoy H. Wieringa B. Martens G. J. M. (2017). The SH-SY5Y cell line in Parkinson's disease research: a systematic review. Mol. Neurodegener. 12, 10. 10.1186/s13024-017-0149-0 PMC5259880 28118852

[B238] Xie W. Chung K. K. (2012). Alpha-synuclein impairs normal dynamics of mitochondria in cell and animal models of Parkinson's disease. J. Neurochem. 122, 404–414. 10.1111/j.1471-4159.2012.07769.x 22537068

[B239] Yao K. Zhao Y. F. (2018). Aging modulates microglia phenotypes in neuroinflammation of MPTP-PD mice. Exp. Gerontol. 111, 86–93. 10.1016/j.exger.2018.07.010 30009921

[B240] Yao L. Zhu Z. Wu J. Zhang Y. Zhang H. Sun X. . (2019). MicroRNA-124 regulates the expression of p62/p38 and promotes autophagy in the inflammatory pathogenesis of Parkinson's disease. FASEB J. 33, 8648–8665. 10.1096/fj.201900363R 30995872

[B241] Yavich L. Tanila H. Vepsäläinen S. Jäkälä P. (2004). Role of α-synuclein in presynaptic dopamine recruitment. J. Neurosci. 24, 11165–11170. 10.1523/JNEUROSCI.2559-04.2004 15590933 PMC6730279

[B242] Ye J. Jiang Z. Chen X. Liu M. Li J. Liu N. (2016). Electron transport chain inhibitors induce microglia activation through enhancing mitochondrial reactive oxygen species production. Exp. Cell Res. 340, 315–326. 10.1016/j.yexcr.2015.10.026 26511505

[B243] Yi M. Weaver D. Hajnóczky G. (2004). Control of mitochondrial motility and distribution by the calcium signal: a homeostatic circuit. J. Cell Biol. 167, 661–672. 10.1083/jcb.200406038 15545319 PMC2172592

[B244] Yu Z. Jiang N. Su W. Zhuo Y. (2021). Necroptosis: a novel pathway in neuroinflammation. Front. Pharmacol. 12, 701564. 10.3389/fphar.2021.701564 PMC8311004 34322024

[B245] Zaheer A. Knight S. Zaheer A. Ahrens M. Sahu S. K. Yang B. (2008a). Glia maturation factor overexpression in neuroblastoma cells activates glycogen synthase kinase-3beta and caspase-3. Brain Res. 1190, 206–214. 10.1016/j.brainres.2007.11.011 18054898 PMC2343001

[B246] Zaheer A. Zaheer S. Thangavel R. Wu Y. Sahu S. K. Yang B. (2008b). Glia maturation factor modulates beta-amyloid-induced glial activation, inflammatory cytokine/chemokine production and neuronal damage. Brain Res. 1208, 192–203. 10.1016/j.brainres.2008.02.093 18395194 PMC2587299

[B247] Zhang C. Sajith A. M. Xu X. Jiang J. Phillip Bowen J. Kulkarni A. . (2022). Targeting NLRP3 signaling by a novel-designed sulfonylurea compound for inhibition of microglial inflammation. Bioorg. Med. Chem. 58, 116645. 10.1016/j.bmc.2022.116645 PMC8895276 35151118

[B248] Zhang Y. N. Fan J. K. Gu L. Yang H. M. Zhan S. Q. Zhang H. (2021). Metabotropic glutamate receptor 5 inhibits α-synuclein-induced microglia inflammation to protect from neurotoxicity in Parkinson's disease. J. Neuroinflammation 18, 23. 10.1186/s12974-021-02079-1 33461598 PMC7814625

[B249] Zhao J. Bi W. Xiao S. Lan X. Cheng X. Zhang J. . (2019). Neuroinflammation induced by lipopolysaccharide causes cognitive impairment in mice. Sci. Rep. 9, 5790. 10.1038/s41598-019-42286-8 PMC6453933 30962497

[B250] Zhao Y. F. Qiong Z. Zhang J. F. Lou Z. Y. Zu H. B. Wang Z. G. . (2018). The synergy of aging and LPS exposure in a mouse model of Parkinson's disease. Aging Dis. 9, 785–797. 10.14336/AD.2017.1028 30271656 PMC6147589

[B251] Zhou Y. Lu M. Du R. H. Qiao C. Jiang C. Y. Zhang K. Z. . (2016). MicroRNA-7 targets Nod-like receptor protein 3 inflammasome to modulate neuroinflammation in the pathogenesis of Parkinson's disease. Mol. Neurodegener. 11, 28. 10.1186/s13024-016-0094-3 PMC4833896 27084336

[B252] Zhu J. Hu Z. Han X. Wang D. Jiang Q. Ding J. . (2018). Dopamine D2 receptor restricts astrocytic NLRP3 inflammasome activation via enhancing the interaction of β-arrestin2 and NLRP3. Cell Death Differ. 25, 2037–2049. 10.1038/s41418-018-0127-2 29786071 PMC6219479

[B253] Zhu Y. Chen X. Liu Z. Peng Y. P. Qiu Y. H. (2015). Interleukin-10 protection against lipopolysaccharide-induced neuro-inflammation and neurotoxicity in ventral mesencephalic cultures. Int. J. Mol. Sci. 17, 25. 10.3390/ijms17010025 PMC4730272 26729090

