# Supplementary material for: Pre-clinical Studies Identifying Molecular Pathways of Neuroinflammation in Parkinson's Disease: A Systematic Review
Source: Front Aging Neurosci. 2022 Jul 4;14:855776. doi: 10.3389/fnagi.2022.855776 (PMC9327618; doi:10.3389/fnagi.2022.855776)
Supplement: Supplementary file 1 [file Table_1.DOCX]

|  | (Hong, Cho et al. 2018) | (Neal, Boyle et al. 2018) | (Kim, Ho et al. 2013) | (Kim, Jeong et al. 2016) | (Ikeda-Matsuo, Miyata et al. 2019) | (Sarkar, Nguyen et al. 2020) | (Morales-Garcia, Alonso-Gil et al. 2020) | (Shin, Jeon et al. 2015) | (Jo, Ikram et al. 2019) | (Dauer, Kholodilov et al. 2002) | (Kilpeläinen, Julku et al. 2019) | (Gispert, Del Turco et al. 2003) | (Giasson, Duda et al. 2002) | (Gu, Long et al. 2010) | (Li, Nadanaciva et al. 2013) | (Chesselet, Richter et al. 2012) | (Fleming, Tetreault et al. 2008) | (Przedborski, Tieu et al. 2004) | (Liberatore, Jackson-Lewis et al. 1999) | (Przedborski, Jackson-Lewis et al. 2000) | (Ambrosi, Kustrimovic et al. 2017) | (Theodore, Cao et al. 2008) | (Iba, Kim et al. 2020) | (Brochard, Combadière et al. 2009) | (Subbarayan, Hudson et al. 2020) | (Karikari, McFleder et al. 2022) | (Zhang, Sajith et al. 2022) | (Miklossy, Doudet et al. 2006) | (Mao, Gao et al. 2021) | (Lai, Kim et al. 2022) | (La Vitola, Balducci et al. 2021) | (Zhang, Fan et al. 2021) | (Williams, Schonhoff et al. 2021) | (Trudler, Nazor et al. 2021) | (Sarkar, Dammer et al. 2020) | (Javed, Thangavel et al. 2020) | (Earls, Menees et al. 2019) | (Li, Niu et al. 2019) | (Fernagut, Hutson et al. 2007) | (Panicker, Sarkar et al. 2019) |
| --- | --- | --- | --- | --- | --- | --- | --- | --- | --- | --- | --- | --- | --- | --- | --- | --- | --- | --- | --- | --- | --- | --- | --- | --- | --- | --- | --- | --- | --- | --- | --- | --- | --- | --- | --- | --- | --- | --- | --- | --- |
| 1)Was the allocation  adequately generated and applied? | unclear | unclear | unclear | unclear | unclear | unclear | unclear | unclear | unclear | yes | unclear | unclear | unclear | yes | unclear | unclear | unclear | unclear | yes | unclear | unclear | unclear | unclear | unclear | unclear | unclear | unclear | unclear | yes | unclear | unclear | unclear | no | unclear | unclear | unclear | unclear | unclear | unclear | unclear |
| 2)Were the groups similar at baseline or were they adjusted for confounders in the analysis? | yes | yes | yes | yes | yes | yes | yes | yes | yes | yes | yes | yes | yes | yes | unclear | yes | yes | unclear | yes | yes | yes | yes | no | yes | yes | no | yes | yes | yes | yes | yes | yes | yes | yes | yes | yes | yes | unclear | yes | yes |
| 3) Was the allocation to the different groups adequately concealed during? | no | no | no | no | no | no | no | no | no | yes | no | unclear | unclear | unclear | unclear | unclear | unclear | unclear | yes | unclear | yes | unclear | unclear | unclear | yes | unclear | unclear | unclear | unclear | no | no | no | unclear | unclear | unclear | unclear | unclear | no | no | no |
| 4)Were the animals randomly housed during the experiment? | unclear | unclear | unclear | yes | unclear | unclear | unclear | unclear | unclear | yes | unclear | unclear | unclear | unclear | unclear | unclear | yes | yes | unclear | unclear | unclear | unclear | unclear | yes | unclear | yes | yes | yes | unclear | unclear | unclear | unclear | unclear | unclear | unclear | unclear | unclear | unclear | unclear | unclear |
| 5)Were the caregivers and/or investigators blinded from knowledge which intervention each animal received during the experiment? | unclear | unclear | unclear | yes | unclear | unclear | unclear | unclear | unclear | unclear | unclear | unclear | yes | unclear | unclear | unclear | unclear | no | no | unclear | unclear | unclear | no | unclear | unclear | unclear | unclear | unclear | no | unclear | yes | unclear | unclear | unclear | yes | unclear | unclear | unclear | unclear | unclear |
| 6)Were animals selected at random for outcome assessment? | unclear | unclear | unclear | unclear | yes | unclear | unclear | unclear | unclear | unclear | unclear | unclear | unclear | unclear | unclear | no | unclear | unclear | unclear | unclear | unclear | unclear | unclear | unclear | no | unclear | no | unclear | unclear | unclear | unclear | yes | unclear | unclear | unclear | unclear | unclear | unclear | no | unclear |
| 7)Was the outcome assessor blinded? | yes | yes | yes | yes | unclear | yes | yes | yes | yes | yes | yes | unclear | unclear | yes | unclear | unclear | unclear | yes | yes | yes | yes | yes | unclear | unclear | unclear | yes | no | unclear | yes | yes | yes | yes | yes | no | yes | yes | unclear | yes | yes | yes |
| 8)Were incomplete outcome data adequately addressed? | yes | yes | yes | yes | yes | yes | yes | yes | yes | unclear | yes | unclear | unclear | unclear | unclear | yes | unclear | yes | unclear | yes | unclear | unclear | unclear | unclear | yes | unclear | unclear | yes | unclear | yes | no | yes | yes | yes | yes | yes | unclear | yes | yes | yes |
| 9)Are reports of the study free of selective data reporting? | yes | yes | yes | yes | yes | yes | yes | yes | yes | unclear | yes | yes | yes | yes | unclear | no | yes | unclear | yes | yes | yes | yes | no | unclear | yes | yes | unclear | unclear | yes | yes | yes | yes | yes | yes | yes | yes | yes | yes | yes | yes |
| 10)Was the study apparently free of other problems that could result in high risk of bias? | yes | unclear | unclear | unclear | yes | unclear | unclear | unclear | yes | no | unclear | yes | unclear | yes | yes | yes | yes | yes | unclear | yes | yes | unclear | no | yes | yes | yes | yes | yes | yes | unclear | unclear | unclear | unclear | yes | no | yes | unclear | unclear | unclear | unclear |

Ambrosi, G., et al. (2017). "Complex Changes in the Innate and Adaptive Immunity Accompany Progressive Degeneration of the Nigrostriatal Pathway Induced by Intrastriatal Injection of 6-Hydroxydopamine in the Rat." Neurotox Res **32**(1): 71-81.

Brochard, V., et al. (2009). "Infiltration of CD4+ lymphocytes into the brain contributes to neurodegeneration in a mouse model of Parkinson disease." J Clin Invest **119**(1): 182-192.

Chesselet, M.-F., et al. (2012). "A progressive mouse model of Parkinson's disease: the Thy1-aSyn ("Line 61") mice." Neurotherapeutics : the journal of the American Society for Experimental NeuroTherapeutics **9**(2): 297-314.

Dauer, W., et al. (2002). "Resistance of α-synuclein null mice to the parkinsonian neurotoxin MPTP." Proceedings of the National Academy of Sciences **99**(22): 14524-14529.

Earls, R. H., et al. (2019). "Intrastriatal injection of preformed alpha-synuclein fibrils alters central and peripheral immune cell profiles in non-transgenic mice." J Neuroinflammation **16**(1): 250.

Fernagut, P. O., et al. (2007). "Behavioral and histopathological consequences of paraquat intoxication in mice: effects of alpha-synuclein over-expression." Synapse **61**(12): 991-1001.

Fleming, S. M., et al. (2008). "Olfactory deficits in mice overexpressing human wildtype alpha-synuclein." Eur J Neurosci **28**(2): 247-256.

Giasson, B. I., et al. (2002). "Neuronal α-Synucleinopathy with Severe Movement Disorder in Mice Expressing A53T Human α-Synuclein." Neuron **34**(4): 521-533.

Gispert, S., et al. (2003). "Transgenic mice expressing mutant A53T human alpha-synuclein show neuronal dysfunction in the absence of aggregate formation." Mol Cell Neurosci **24**(2): 419-429.

Gu, X. L., et al. (2010). "Astrocytic expression of Parkinson's disease-related A53T alpha-synuclein causes neurodegeneration in mice." Mol Brain **3**: 12.

Hong, G. U., et al. (2018). "Inflammatory mediators resulting from transglutaminase 2 expressed in mast cells contribute to the development of Parkinson's disease in a mouse model." Toxicol Appl Pharmacol **358**: 10-22.

Iba, M., et al. (2020). "Neuroinflammation is associated with infiltration of T cells in Lewy body disease and α-synuclein transgenic models." J Neuroinflammation **17**(1): 214.

Ikeda-Matsuo, Y., et al. (2019). "Microsomal prostaglandin E synthase-1 is a critical factor in dopaminergic neurodegeneration in Parkinson's disease." Neurobiol Dis **124**: 81-92.

Javed, H., et al. (2020). "NLRP3 inflammasome and glia maturation factor coordinately regulate neuroinflammation and neuronal loss in MPTP mouse model of Parkinson's disease." Int Immunopharmacol **83**: 106441.

Jo, M. G., et al. (2019). "Gintonin Mitigates MPTP-Induced Loss of Nigrostriatal Dopaminergic Neurons and Accumulation of α-Synuclein via the Nrf2/HO-1 Pathway." Mol Neurobiol **56**(1): 39-55.

Karikari, A. A., et al. (2022). "Neurodegeneration by α-synuclein-specific T cells in AAV-A53T-α-synuclein Parkinson's disease mice." Brain Behav Immun **101**: 194-210.

Kilpeläinen, T., et al. (2019). "Behavioural and dopaminergic changes in double mutated human A30P*A53T alpha-synuclein transgenic mouse model of Parkinson´s disease." Scientific Reports **9**(1): 17382.

Kim, B. W., et al. (2016). "Pathogenic Upregulation of Glial Lipocalin-2 in the Parkinsonian Dopaminergic System." J Neurosci **36**(20): 5608-5622.

Kim, C., et al. (2013). "Neuron-released oligomeric α-synuclein is an endogenous agonist of TLR2 for paracrine activation of microglia." Nat Commun **4**: 1562.

La Vitola, P., et al. (2021). "Peripheral inflammation exacerbates α-synuclein toxicity and neuropathology in Parkinson's models." Neuropathol Appl Neurobiol **47**(1): 43-60.

Lai, T. T., et al. (2022). "Evidence of Inflammation in Parkinson's Disease and Its Contribution to Synucleinopathy." J Mov Disord **15**(1): 1-14.

Li, L., et al. (2013). "Human A53T α-synuclein causes reversible deficits in mitochondrial function and dynamics in primary mouse cortical neurons." PLoS One **8**(12): e85815.

Li, Y., et al. (2019). "CXCL12 is involved in α-synuclein-triggered neuroinflammation of Parkinson's disease." J Neuroinflammation **16**(1): 263.

Liberatore, G. T., et al. (1999). "Inducible nitric oxide synthase stimulates dopaminergic neurodegeneration in the MPTP model of Parkinson disease." Nat Med **5**(12): 1403-1409.

Mao, J., et al. (2021). "Lipoic acid alleviates LPS‑evoked PC12 cell damage by targeting p53 and inactivating the NF‑κB pathway." Acta Neurobiol Exp (Wars) **81**(4): 375-385.

Miklossy, J., et al. (2006). "Role of ICAM-1 in persisting inflammation in Parkinson disease and MPTP monkeys." Exp Neurol **197**(2): 275-283.

Morales-Garcia, J. A., et al. (2020). "Phosphodiesterase 7 Regulation in Cellular and Rodent Models of Parkinson's Disease." Mol Neurobiol **57**(2): 806-822.

Neal, M. L., et al. (2018). "The glycoprotein GPNMB attenuates astrocyte inflammatory responses through the CD44 receptor." J Neuroinflammation **15**(1): 73.

Panicker, N., et al. (2019). "Fyn kinase regulates misfolded α-synuclein uptake and NLRP3 inflammasome activation in microglia." J Exp Med **216**(6): 1411-1430.

Przedborski, S., et al. (2000). "The parkinsonian toxin MPTP: action and mechanism." Restor Neurol Neurosci **16**(2): 135-142.

Przedborski, S., et al. (2004). "MPTP as a mitochondrial neurotoxic model of Parkinson's disease." J Bioenerg Biomembr **36**(4): 375-379.

Sarkar, S., et al. (2020). "Molecular Signatures of Neuroinflammation Induced by αSynuclein Aggregates in Microglial Cells." Front Immunol **11**: 33.

Sarkar, S., et al. (2020). "Kv1.3 modulates neuroinflammation and neurodegeneration in Parkinson's disease." J Clin Invest **130**(8): 4195-4212.

Shin, W. H., et al. (2015). "Induction of microglial toll-like receptor 4 by prothrombin kringle-2: a potential pathogenic mechanism in Parkinson's disease." Sci Rep **5**: 14764.

Subbarayan, M. S., et al. (2020). "T cell infiltration and upregulation of MHCII in microglia leads to accelerated neuronal loss in an α-synuclein rat model of Parkinson's disease." J Neuroinflammation **17**(1): 242.

Theodore, S., et al. (2008). "Targeted overexpression of human alpha-synuclein triggers microglial activation and an adaptive immune response in a mouse model of Parkinson disease." J Neuropathol Exp Neurol **67**(12): 1149-1158.

Trudler, D., et al. (2021). "Soluble α-synuclein-antibody complexes activate the NLRP3 inflammasome in hiPSC-derived microglia." Proc Natl Acad Sci U S A **118**(15).

Williams, G. P., et al. (2021). "CD4 T cells mediate brain inflammation and neurodegeneration in a mouse model of Parkinson's disease." Brain **144**(7): 2047-2059.

Zhang, C., et al. (2022). "Targeting NLRP3 signaling by a novel-designed sulfonylurea compound for inhibition of microglial inflammation." Bioorg Med Chem **58**: 116645.

Zhang, Y. N., et al. (2021). "Metabotropic glutamate receptor 5 inhibits α-synuclein-induced microglia inflammation to protect from neurotoxicity in Parkinson's disease." J Neuroinflammation **18**(1): 23.
